# Hong–Ou–Mandel interference of more than ten indistinguishable atoms

**DOI:** 10.1038/s41567-026-03302-7

**Published:** 2026-06-01

**Authors:** Martin Quensen, Mareike Hetzel, Luis Santos, Augusto Smerzi, Géza Tóth, Luca Pezzè, Carsten Klempt

**Affiliations:** 1 Institut für Quantenoptik, Leibniz Universität Hannover, Hannover, Germany; 2 Institut für Satellitengeodäsie und Inertialsensorik (DLR-SI), Deutsches Zentrum für Luft- und Raumfahrt e.V. (DLR), Hannover, Germany; 3 Instituto Nazionale di Ottica, Consiglio Nazionale delle Ricerche (INO-CNR), Florence, Italy; 4 European Laboratory for Nonlinear Spectroscopy (LENS), Sesto Fiorentino, Italy; 5 Department of Theoretical Physics, University of the Basque Country UPV/EHU, Bilbao, Spain; 6 EHU Quantum Center, University of the Basque Country UPV/EHU, Leioa, Spain; 7 IKERBASQUE, Basque Foundation for Science, Bilbao, Spain; 8 Donostia International Physics Center (DIPC), San Sebastián, Spain; 9 HUN-REN Wigner Research Centre for Physics, Budapest, Hungary

**Keywords:** Quantum metrology, Single photons and quantum effects

## Abstract

When two indistinguishable bosons interfere at a beam splitter, they exit through the same output port. This quantum effect, known as Hong–Ou–Mandel interference, underpins many protocols in quantum information. It also generalizes to larger numbers of identical particles, for which interference produces characteristic many-body patterns. So far, experiments have observed the many-particle regime mainly in photonic platforms with unavoidable loss, and atomic realizations have remained challenging. Here we demonstrate Hong–Ou–Mandel interference with up to 12 indistinguishable neutral atoms in a system with negligible loss. Single-particle counting reveals parity oscillations, a bunching envelope and genuine multipartite entanglement, which are defining features of the multiparticle Hong–Ou–Mandel effect. Using the generated quantum states, we demonstrate metrological sensitivities that scale with the number of particles according to the Heisenberg limit. Our technique can be extended to larger ensembles, with wide-ranging applications from high-precision atom interferometry to multiparticle Bell tests.

## Abstract (web summary)

Hong–Ou–Mandel interference has been difficult to extend to ensembles of many massive particles. Measurements on up to 12 neutral atoms now exhibit characteristic multiparticle interference pattern with single-atom resolution and negligible loss.

## Main

**Fig. 1:**
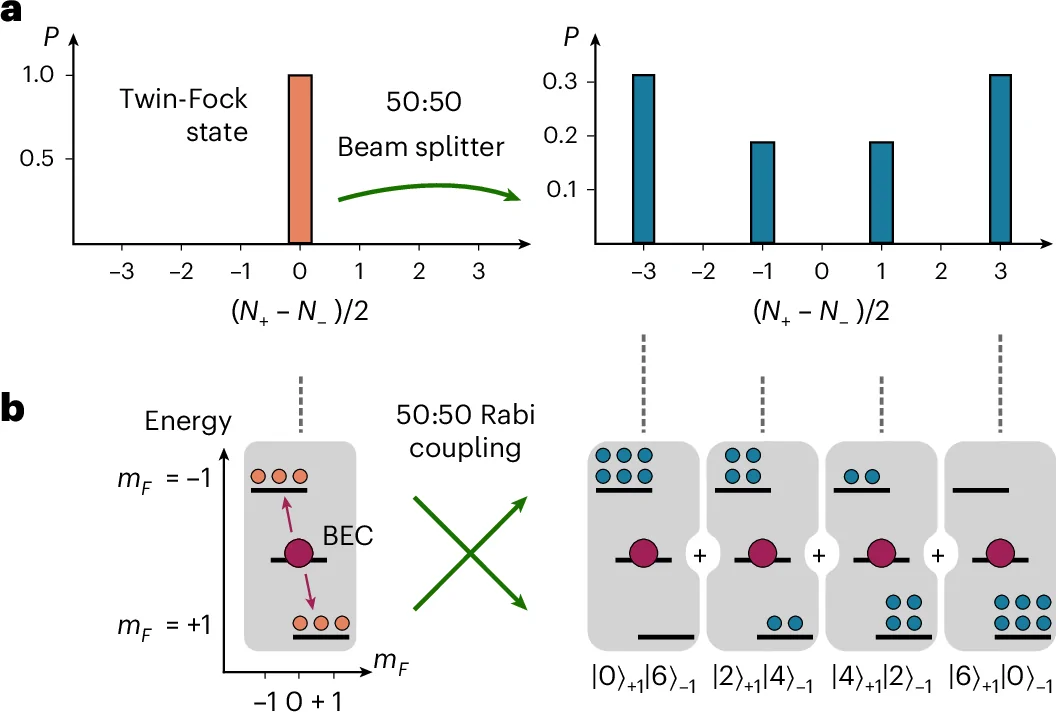
Multiparticle HOM interference. **a**, If *N*_+_ = 3 and *N*_−_ = 3 identical particles in a twin-Fock state (left panel) interfere on a 50:50 beam splitter, the resulting state shows a characteristic distribution with only even numbers of particles at the two output ports (right). The broad distribution of the number difference (*N*_+_ − *N*_−_)/2 has maximal contributions for all particles bunching at the same output port. **b**, Spin-changing collisions within a BEC in the Zeeman level $\left(F,{m}_{F}\right)=\left(1,0\right)$ enable the creation of such twin-Fock states in the two levels $\left(1,\pm 1\right)$ (left). Here, *F* = 1, 2 denotes the quantum number of the total atomic angular momentum and *m*_*F*_= −*F*, −*F*+1, …, *F* is the magnetic quantum number. A 50:50 Rabi coupling of these two levels is equivalent to a beam splitter and realizes the same distribution of only even numbers.

Particle indistinguishability leads to striking quantum interference phenomena, as first demonstrated with a pair of photons in the landmark experiment by Hong, Ou and Mandel^1^. The absence of simultaneous detection events at the two output ports of a balanced beam splitter serves as a central figure of merit for the generation of indistinguishable pairs. The concept extends to bimodal states with a larger number of particles (Fig. 1a) and their multiparticle interference. If the same number of indistinguishable particles (a twin-Fock state) enter the two inputs of a 50:50 beam splitter, the output state exhibits a characteristic distribution of only even numbers with a bunching envelope^2^. The Hong–Ou–Mandel (HOM) effect plays a key role in both quantum metrology^3^ and quantum information and has become a textbook example of an interference phenomenon that cannot be explained by a semiclassical theory^4^. The twin-Fock input states of the HOM interferometer are highly non-classical quantum states that are particle entangled according to the definition of refs. ^5,6^. They offer enhanced sensitivity for interferometric applications^7^, as they surpass the standard quantum limit and enable Heisenberg scaling^8^.

In optics, spontaneous parametric downconversion (SPDC) is the prevalent method for creating indistinguishable photon pairs. It is integral to quantum cryptography^9^, quantum computing^10^, quantum metrology^11,12^ and fundamental tests of quantum mechanics^13^. By driving SPDC to higher numbers of photons, multiphoton interference of four^14,15^, six^16–19^ and eight photons^20^ has been observed and entanglement-enhanced sensitivities have been derived. Although photon detectors have been developed with number-resolving capabilities extending to dozens of photons^21^, photonic systems still encounter major challenges, such as unwanted higher multiphoton components, partial distinguishability and inevitable photon loss^22^; these imperfections affect the measured quantities^23^ and compromise the comparison of model-free quantum mechanical predictions and experimental observations. Furthermore, these effects strongly constrain the scaling to larger numbers of particles^24^.

The generation of indistinguishable pairs has also been explored in other systems^25^. HOM interference has been demonstrated with microwave photons^26^, ions^27^, Rydberg atoms^28^, and even between photons and atomic magnons^29^. Ultracold atomic systems enable HOM realizations through tunnel coupling in atomic tweezers^30^ or in optical lattices^31–33^. However, in these systems, multiparticle bosonic interference has been limited to multimode settings^34,35^. A direct extension of the original HOM interference from two particles in two modes to *N* particles in two modes has not been reported. Below, we refer to this setting as multiparticle HOM interference.

A scaling to and analysis of HOM interference for larger numbers of particles is outstanding. We realize HOM interference with up to 12 indistinguishable neutral atoms in a system with negligible loss and single-particle-resolving detection. The HOM interference relies on the high-fidelity creation and detection of twin-Fock states generated by spin-changing collisions in Bose–Einstein condensates (BECs). We analyse these states by evaluating parity observables, by measuring squeezing parameters as low as −15.4(10) dB and by verifying close-to-optimal multiparticle entanglement. We show that the created quantum state can be employed for entanglement-enhanced interferometry, and we observe a Heisenberg scaling of the Fisher information of the states for up to 12 atoms (corresponding to 6.4(8) dB beyond the classical bound).

## Pair creation in spinor BECs and single-atom counting

Indistinguishable ultracold atoms in a gaseous BEC with a spin degree of freedom represent a paradigmatic many-particle quantum system near absolute zero temperature. A BEC of atoms with a total spin *F* = 1 shows a ground-state phase diagram characterized by three quantum phases^36–38^ with phase transitions driven by a pair creation process. Although this process generates states with equal numbers of particles akin to SPDC^39,40^, the correlations at the single-particle level have previously been witnessed only indirectly. Direct detection has been hindered by detection noise, typically on the order of a few atoms, stemming from technical noise in the recorded absorption or fluorescence signals. With an improved fluorescence detection set-up, it has been possible to observe pair creation with a counting noise of 1.6 atoms^41^, which is close to but not beyond the single-atom-resolution threshold. In our experiments, we reached a counting resolution of 0.2 atoms, which enables the direct observation of HOM interference in spinor BECs.

## Experimental procedure

**Extended Data Fig. 1:**
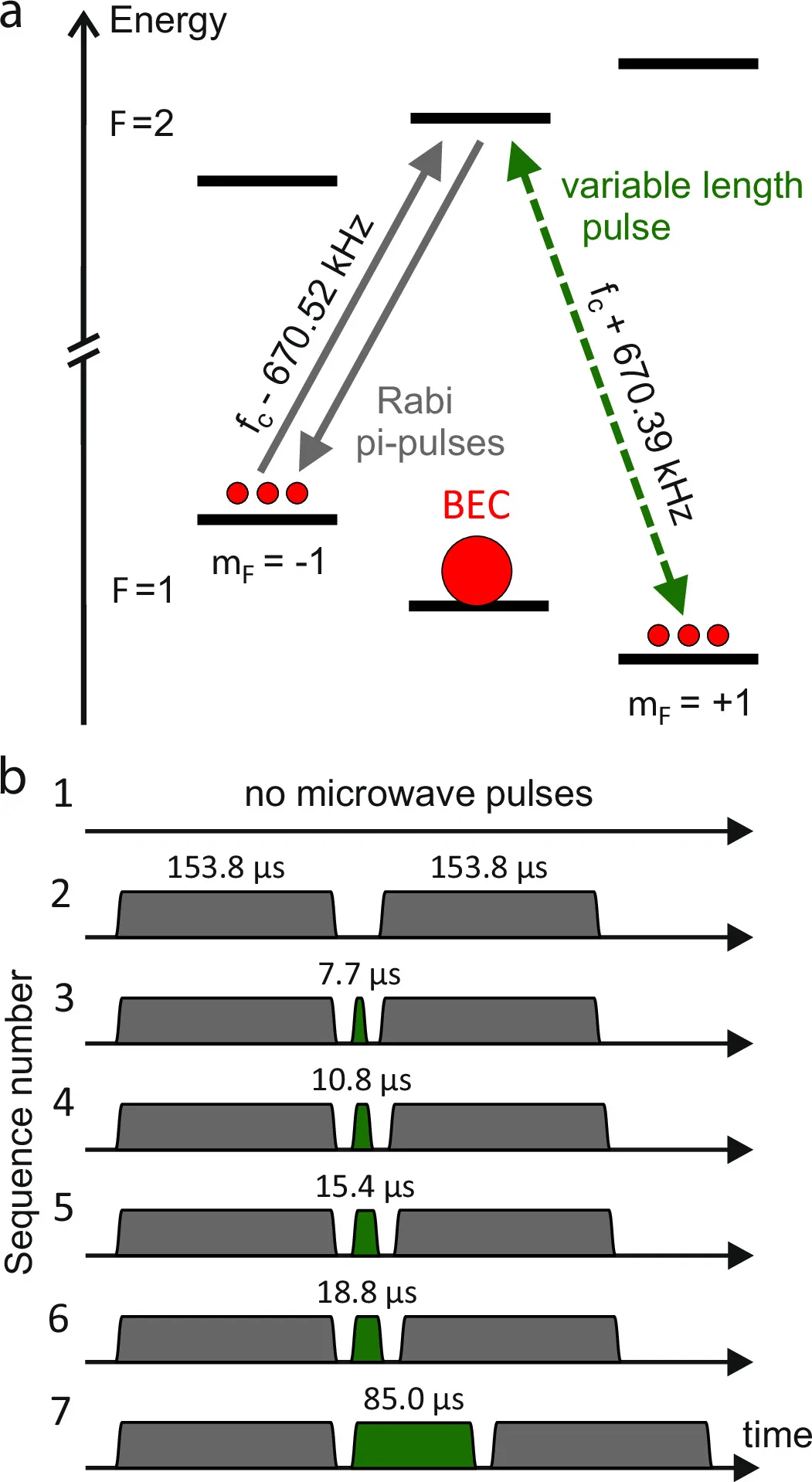
Tunable mode coupling via microwave Rabi pulses. **a**, A sequence of three microwave pulses with frequencies around *f*_*c*_ = 6.835 GHz is employed for a variable Rabi coupling of the twin-Fock modes $\left(1,\pm 1\right)$. The coupling strength is controlled by the duration of the second Rabi pulse. **b**, For the measurements presented here, a total of seven different microwave sequences were repeated 3816 times each. Sequence 1 and 2 both result in unmodified two-mode squeezed vacuum states. Figures 2d and 3a show the data from sequence 2. Sequences 3 to 6 constitute small rotations of the twin-Fock states, employed for the results presented in Fig. 5. Sequence 7 provides the data for the Hong-Ou-Mandel coupling, presented in Fig. 2e and Fig. 3b, and utilised for analysis results given in Fig. 3d,e and Fig. 4.

We generated rubidium BECs of 250 atoms in a crossed-beam optical dipole trap with waists of 35 μm and 5 μm, respectively^42^. The atoms were predominantly prepared in the Zeeman level $\left(F,{m}_{F}\right)=\left(1,0\right)$ by spin-distillation cooling and purified during a cleaning and compression sequence (Methods). When spin dynamics is initiated by tuning the quadratic Zeeman energy *q* for a finite time *t* = 120 ms, spin-changing collisions generate particles in pairs (Fig. 1b), leading to a two-mode squeezed vacuum (TMSV) state^43^:

1 $$\left|\xi \,\right\rangle =\mathop{\sum }\limits_{n=0}^{\infty }\frac{{(-{\rm{i}}\,\tanh \,\xi \,)}^{n}}{\cosh \,\xi }{\left|n\right\rangle }_{+1}{\left|n\right\rangle }_{-1},$$

where *ξ* = *Ωt* is the squeezing parameter, which depends on the spin-dynamics rate *Ω* = 2*π* × 2.2 Hz. The notation ${\left|n\right\rangle }_{+1}{\left|m\right\rangle }_{-1}$ denotes a two-mode Fock state with *n* atoms in the Zeeman level $\left(1,+1\right)$ and *m* atoms in the Zeeman level $\left(1,-1\right)$, respectively. The TMSV state constitutes a coherent superposition of twin-Fock states with equal numbers of particles in the two modes. As in SPDC, the final measurement of the mode occupation numbers will determine the total number of particles *N* in the state. Because states with different numbers of particles do not interfere, we can treat the quantum state as a single twin-Fock state before the HOM interference. Interference is initiated by coupling the levels $\left(1,\pm 1\right)$ through a sequence of resonant microwave pulses, effectively achieving a 50:50 Rabi coupling (Methods and Extended Data Fig. 1). The coupling yields an output state according to

2 $${\left|n\right\rangle }_{+1}{\left|n\right\rangle }_{-1}\to \mathop{\sum }\limits_{k=0}^{n}{c}_{k}{\left|2k\right\rangle }_{+1}{\left|2n-2k\right\rangle }_{-1},$$

where the coefficients *c*_*k*_ follow a discrete arcsine distribution^2^:

3 $${\left|{c}_{k}\right|}^{2}=\left(\begin{array}{c}2k\\ k\end{array}\right)\left(\begin{array}{c}2n-2k\\ n-k\end{array}\right){\left(\frac{1}{2}\right)}^{2n}.$$

There is a characteristic occupation with only even numbers of atoms and an enhanced probability of extremal states (${\left|N\right\rangle }_{+1}{\left|0\right\rangle }_{-1}$ and ${\left|0\right\rangle }_{+1}{\left|N\right\rangle }_{-1}$) in which most bosons occupy the same mode (bunching). After coupling, this twin-Fock state is also called a Holland–Burnett state^3^. Demonstrating the absence of odd occupation numbers in the final state constitutes a primary goal of our experiments and requires a single-atom-resolved counting of the occupation numbers in the levels $\left(1,\pm 1\right)$.

## Accurately counting atoms

**Fig. 2:**
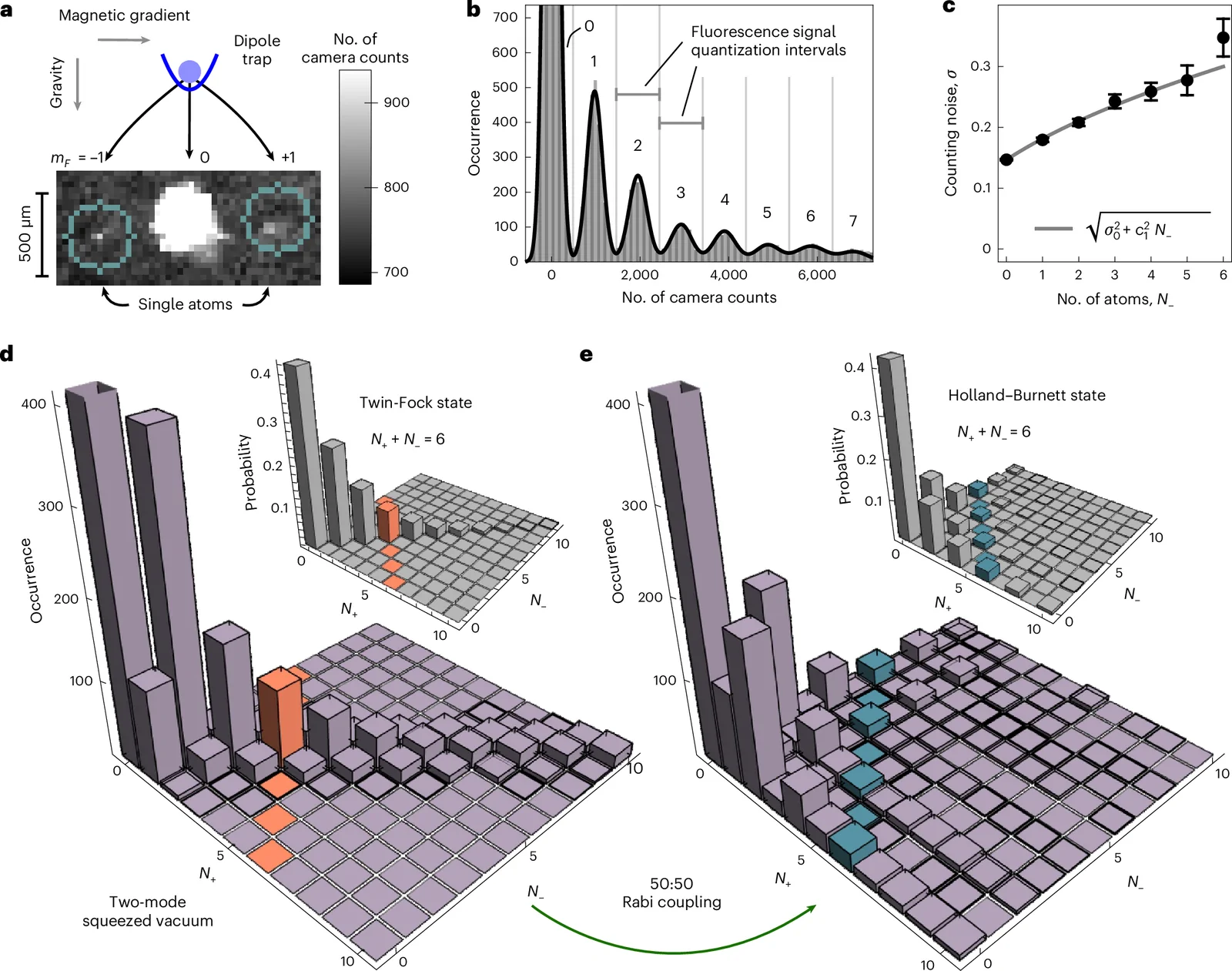
Accurate counting of atoms in a TMSV state. **a**, A TMSV state is created in the levels $\left(1,\pm 1\right)$ in an optical dipole trap. For counting, the atoms in these levels are spatially separated by a strong magnetic field gradient during free fall. They are illuminated with near-resonant laser light and imaged on a CCD camera. The displayed picture shows an exemplary fluorescence signal from two single atoms and the regions of interest used for signal summation (teal circles). **b**, The exemplary histogram of the measured camera signal of the *m*_*F*_ = −1 level (after subtracting two systematic effects; Methods and Extended Data Fig. 3) from 26,712 repeated measurements features distinct peaks, demonstrating single-atom-resolved counting. Gaussian fits provide a calibration of 975.8(16) counts per atom (fitted peak spacing). **c**, The atom counting noise *σ* (fitted peak widths) is well below the single-atom level (Methods). The *m*_*F*_ = +1 data are displayed in Extended Data Fig. 2b,c. The error bars represent the standard error of the fitting parameters. The demonstrated single-atom-resolved counting capability constitutes our main technical breakthrough. It enables the observation of atomic HOM interference. **d**, The detection method was used to obtain the distribution of the numbers of atoms for the TMSV state. The preferential occupation of equal numbers of atoms *N*_+_ = *N*_−_ (the diagonal of the histogram) is a characteristic of the ideal state (inset). **e**, After a 50:50 coupling of the two levels, the resulting histogram shows the characteristic chequerboard pattern with combinations of even numbers only (inset: ideal state). Each subspace for a selected total number of atoms (highlighted in orange and blue for *N* = 6) presents a twin-Fock state before (**d**) and a Holland–Burnett state after (**e**) HOM coupling. Each histogram shows a dataset with 3,816 repetitions. Source data

**Extended Data Fig. 2:**
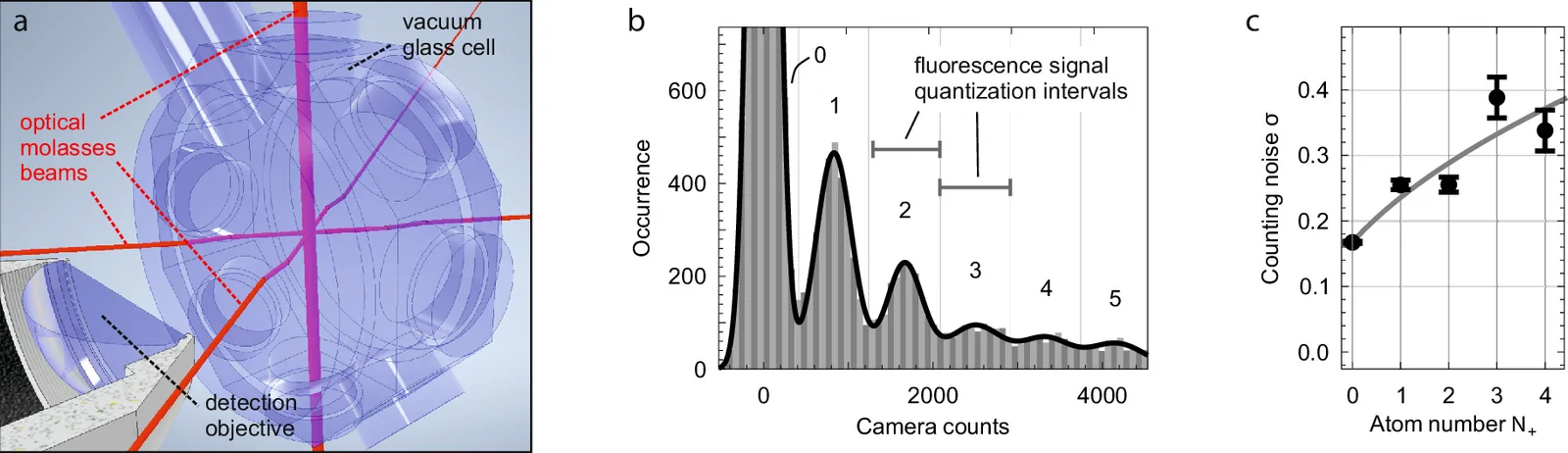
Optical molasses configuration and detection resolution for *m*_*F*_ = + 1. **a**, Six red-detuned beams in molasses configuration illuminate the atomic ensemble for detection. The fluorescence signal is captured by a detection objective and imaged onto a CCD camera. **b**, Histogram of the measured atomic signal of the *m*_*F*_ = + 1 mode for all 26712 recorded measurements (after subtraction of two systematic effects, see Extended Data Fig. 3). The distinct peaks demonstrate the single-atom-resolved counting capability. Gaussian fits yield an atomic signal of 832.5(34) cts/atom (fitted peak spacing). **c**, The counting noise is quantified as the widths *σ*_*N*_ of the Gaussian peaks. The slightly worse performance compared to the *m*_*F*_ = − 1 mode is explained by a less optimal beam intensity balance at the position of the atoms after the spatial separation. Error bars represent the standard error of the obtained fit parameters. Source data

Single-atom-resolved counting is achieved using a fluorescence detection set-up^44–47^, which here operates in the light field configuration of optical molasses (Methods and Extended Data Fig. 2a). The set-up consists of six intersecting laser beams with millimetre-sized diameters, red-detuned by 2π × 6 MHz from the Rb D2 cooling transition. The fluorescence light from the atoms is imaged onto a CCD camera by a high-numerical-aperture lens system. The detuning enables a long illumination time of 4.2 ms due to continuous optical cooling, and the small beam diameters minimize stray light at our illumination intensities of ~3.6 mW cm^−^^2^. By spatially separating the atoms in the three Zeeman levels $\left(1,-1/0/+1\right)$ with a magnetic field gradient before illumination (Fig. 2a), the detection becomes mode-resolved. A single atom in $\left(1,\pm 1\right)$ caused a signal of approximately 900 photoelectron counts on our CCD camera, whereas the recorded background noise was significantly lower, with a standard deviation of less than 0.17 atoms. The quantization of the camera signal to integer numbers of atoms (Fig. 2b) demonstrates the desired single-atom-resolving counting capability. As fluorescence signals of dilute atomic gases do not exhibit any saturation effects^44^, which we checked for our system in a slightly modified, not mode-resolving configuration^47^ for more than 100 atoms, we can extrapolate the quantization intervals linearly to larger numbers of atoms. The counting noise (Fig. 2c) was dominated by the shot noise of the background light and camera noise for small numbers of atoms, and increased due to residual particle movement during the illumination (Methods). We analysed the images so that we could assign integer numbers of atoms *N*_−_, *N*_0_ and *N*_+_ to the three Zeeman levels for each experimental realization.

## Direct observation of many-particle HOM interference

First, the numbers of atoms were recorded for the generated TMSV state. The histogram in Fig. 2d shows how often a specific combination of numbers of atoms *N*_−_ and *N*_+_ was observed during a measurement run with 3,816 identical repetitions. It shows a dominant population of the twin-Fock states with equal numbers of atoms on the diagonal. There are minor off-diagonal contributions arising mainly from non-zero probabilities for miscounting. Further contributions are from unwanted state transfers during the spatial separation (main noise source for *N* < 3), and losses before detection. A model of the noise contributions is provided in Methods. The spin-dynamics rate *Ω* underwent small fluctuations that emerged from variations in the numbers of atoms in the BEC and the dipole trap configuration. Consequently, the distribution of the total number of atoms in the twin-Fock modes slightly deviated from the exponential distribution of equation (1). The distribution in the subspace of a fixed, even number of atoms *N* = *N*_+_ + *N*_−_ (highlighted for *N* = 6) indicates that the spin-changing collisions in our system served as a high-fidelity source of atomic twin-Fock states. HOM interference, realized by a 50:50 Rabi coupling, fully reshaped the population distribution in accordance with equation (3) (Fig. 2e). Detection events near the diagonal region were then least likely.

**Fig. 3:**
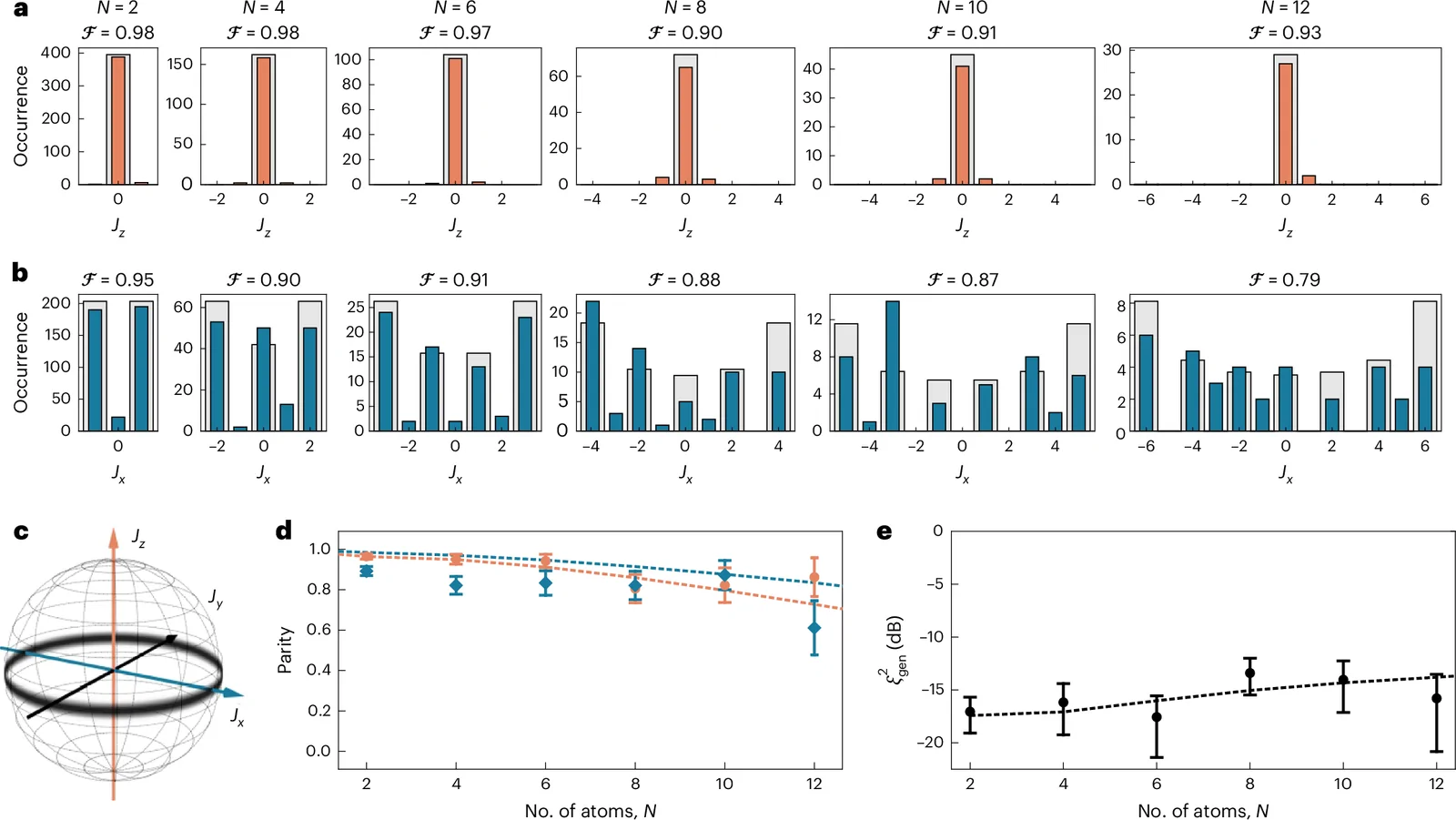
Demonstration of multiparticle HOM interference. **a**,**b**, The observed counting statistics before (**a**) and after (**b**) HOM interference closely match those of the ideal states (grey bars), with fidelities ${\mathcal{F}} > 0.87$ for up to *N* = 10 atoms (${\mathcal{F}}=0.79$ for *N* = 12 atoms after coupling). **c**, These two measurements constitute the observation of *J*_*z*_ and *J*_*x*_ of the initially prepared twin-Fock state, respectively, as illustrated by the Husimi distributions on the generalized Bloch sphere. **d**, The observed parity signals remain near the maximum values of ±1 and indicate the clear suppression of odd occupation numbers after interference. This demonstrates the main characteristic of the HOM effect for up to *N* = 12 particles. **e**, The almost vanishing variance before HOM interference and the near maximum spread after coupling result in generalized squeezing parameters with an average value as low as −15.4(10) dB. The mean values in **d** and **e** were calculated from the counting statistics displayed in **a** and **b**. Error bars denote standard errors of the mean (**d**) and propagated standard errors of the mean (**e**). The dashed lines depict the predictions from our noise model (Methods). Source data

The non-classical features of the detected quantum states are highlighted in the distributions for selected total numbers of atoms *N* (Fig. 3). The data are expressed in terms of the total angular momentum ${\mathbf{J}}={\sum }_{n=1\,}^{N}{\bf{j}}^{(n)}$, defined as the sum of the spin-1/2 operators ${\bf{j}}^{(n)}$ of the *n*th atom in the two levels $\left(1,\pm 1\right)$. Note that a detection error by a single particle does not deteriorate these distributions, as the recorded total number of atoms *N* becomes odd and the event is discarded. All the following analyses are based exclusively on the directly observed population statistics, without incorporating any assumptions about underlying noise contributions.

Figure 3a shows the difference in the number of atoms *J*_*z*_ = (*N*_+_ − *N*_−_)/2 before the HOM interference. The observed twin-Fock states remain very close to the ideal state with *J*_*z*_ = 0, even for *N* = 12 atoms, with fidelities ${\mathcal{F}} > 0.9$. Figure 3b presents the number difference after HOM coupling, which is equivalent to a measurement of *J*_*x*_ of the twin-Fock input state. *J*_*x*_ and *J*_*y*_ must be identical due to the symmetry of the state and the absence of a phase relation between the atoms and the applied microwave pulses. The fidelities of the states after HOM interference, calculated as ${\mathcal{F}}={\left({\sum }_{k=0}^{n}\sqrt{{p}_{k}}\left|{c}_{k}\right|\right)}^{2}$ with *p*_*k*_ the measured probabilities and *c*_*k*_ the coefficients from equation (3), remain as high as 0.87 for up to 10 atoms (${\mathcal{F}}=0.79$ for 12 atoms). These states show a large suppression of odd-numbered mode occupations (Fig. 3d), which can be quantified by the parity operator *Π*_*x*_, where ${\varPi }_{l}={(-1)}^{N/2-{J}_{l}}$ signals if the state exhibits even (*Π*_*l*_ = 1) or odd (*Π*_*l*_ = − 1) occupation numbers before (*l* = *z*) or after (*l* = *x*) HOM interference. We can directly assess the parity observable thanks to our number-resolved detection. Although parity oscillations have been observed with neutral atoms in optical lattices^32^, trapped ions^48^ and Rydberg atoms^49^, our technique, using BECs, offers a particularly promising route to scaling to larger numbers of particles. We obtained values beyond ±0.8 for up to 10 atoms and 0.6 for 12 atoms, revealing a key characteristic of the multiparticle HOM effect. The very low variances $\Delta {J}_{z}^{2} < 0.1$ of the twin-Fock states and the large spread of *J*_*x*,*y*_ after HOM interference led to generalized squeezing parameters ${\xi }_{\mathrm{gen}}^{2}=(N-1)\frac{\Delta {J}_{z}^{2}}{\left\langle \,{J}_{x}^{2}+{J}_{y}^{2}\right\rangle -N/2}$ (refs. ^50,51^) as low as −15.4(10) dB on average (Fig. 3e). Although we did not directly observe a deterioration of ${\xi }_{{\rm{gen}}}^{2}$ for increasing numbers of atoms, our noise model (illustrated by the dashed lines) predicts a moderate decline.

Next, we evaluate the quality of the performed HOM interference further by extracting the multiparticle entanglement of the involved quantum state from the recorded data.

## Multiparticle entanglement

**Fig. 4:**
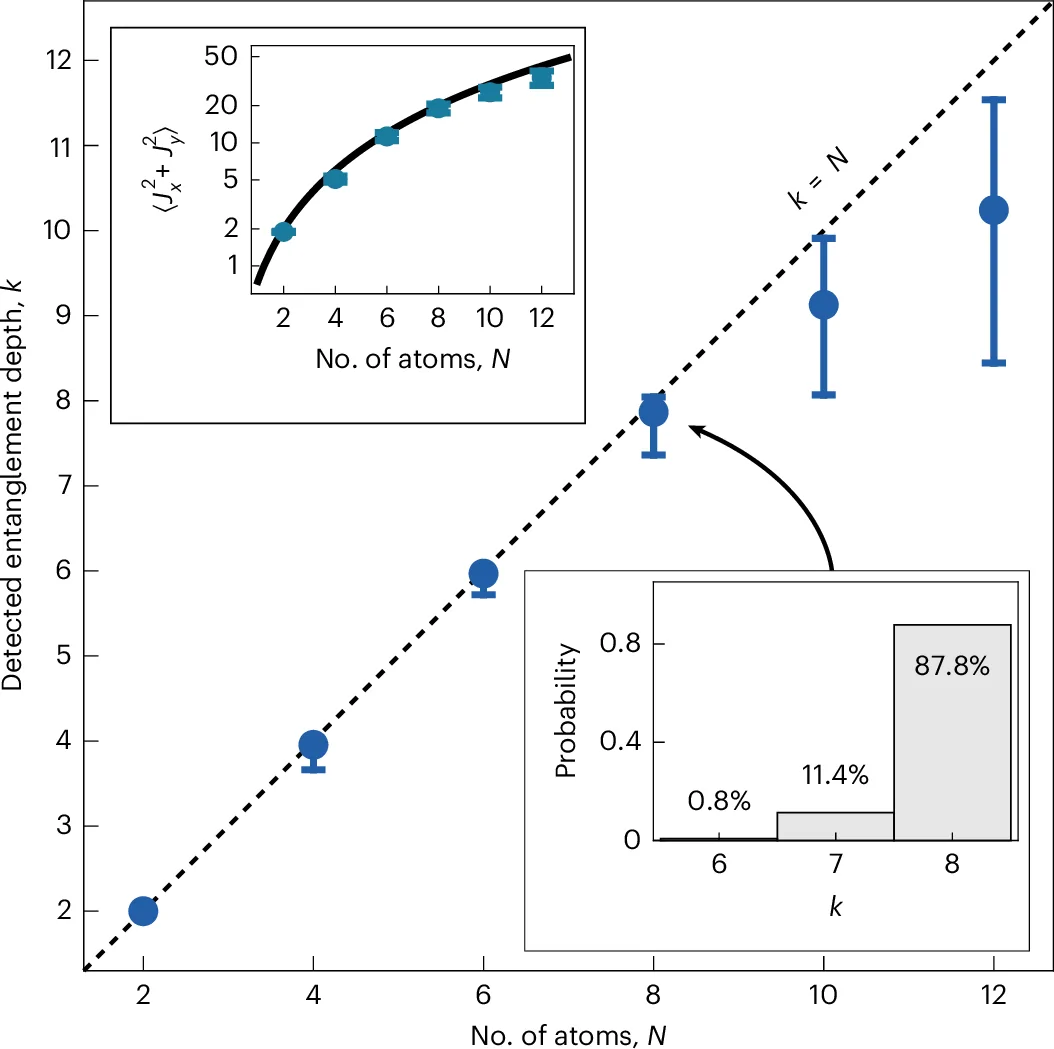
Multiparticle entanglement. The minimal number of entangled atoms *k*, which is compatible with the measured data according to equation (4), demonstrates full genuine multiparticle entanglement for up to *N* = 8 atoms. Top left inset: measured (blue dots) and ideal (black solid line) width $\langle {J}_{x}^{2}+{J}_{y}^{2}\rangle$ of the occupation number difference after HOM interference. The data in the figure represent mean values derived from the counting statistics shown in Fig. 3a,b. The error bars in the inset denote the standard error of the mean, while those in the main figure represent asymmetric standard deviations calculated via the Monte Carlo resampling approach (Methods), which illustrates the uncertainty in the computed distributions of the entanglement depth *k*, depicted here for *N* = 8 (bottom right inset). Source data

Although the *J*_*z*_ measurement without HOM coupling tests the equality of the numbers of particles, the measurement of *J*_*x*,*y*_ after HOM coupling tests the coherence and the indistinguishability of the input state. From these measurements, it is possible to quantify the multiparticle entanglement of the created twin-Fock states. This entanglement is quantified by the entanglement depth, defined as the number of particles in the largest non-separable subset of the quantum state (Methods). We evaluated a lower limit for the entanglement depth from the recorded data. The top inset of Fig. 4 shows the evaluation of $\langle {J}_{x}^{2}+{J}_{y}^{2}\rangle$ as a function of the total number of particles (Extended Data Table 1). The large fluctuations of $\langle {J}_{x}^{2}+{J}_{y}^{2}\rangle$ are close to the optimal value of $\frac{N}{2}(\frac{N}{2}+1)$. The slight deterioration for larger numbers of atoms is attributed to counting noise, loss and fluctuations in the microwave transfer pulses.

From these measurements, we extracted the entanglement depth according to a new criterion (Methods) that is based on the parity *Π*_*z*_:

4 $$\langle \,{J}_{x}^{2}+{J}_{y}^{2}\rangle +\frac{k(N-k)}{2}| \langle {\it\varPi }_{z}\rangle | \le \frac{N}{2}\left(\frac{N}{2}+1\right).$$

The parity directly probes *N*-body correlations within the ensemble. If equation (4) is violated for some *k* ≥ *N*/2, the entanglement depth is at least (*k* + 1). In Fig. 4, the experimental data for up to *N* = 8 atoms demonstrate full *N*-particle entanglement. For larger numbers, the certified entanglement is not maximal but is not fewer than 10 particles in the *N* = 12 case with 68% confidence (Extended Data Table 2). Methods contains a verification of the extracted entanglement depth based on an alternative criterion^50,52^ (Extended Data Fig. 4). It also provides an entanglement proof for the combined state for all *N* ≤ 12, including odd *N*, with a criterion for fluctuating numbers of particles. Here we obtain an entanglement witness value of −1.205(44), which is 27 standard deviations beyond the classical bound of 0. The presented single-atom-resolved counting capabilities enable entanglement certification and quantification that underline the quality and quantum coherence of the generated mesoscopic twin-Fock states.

## Metrological sensitivity

The observed high-fidelity state creation, manipulation and detection can be exploited for interferometric applications. Twin-Fock states can be used to achieve phase sensitivities close to the ultimate Heisenberg limit in systems with a single-atom-resolved detection^7^. We quantified the achievable sensitivity of our *N*-particle twin-Fock states with the Fisher information *F*_*N*_. The Fisher information is related to the Cramér–Rao bound $\Delta {\theta }_{{\rm{CRB}}}=1/\sqrt{{F}_{N}}$ for estimating a Rabi rotation of angle *θ* (ref. ^8^). By following the Hellinger method^53^, we extracted *F*_*N*_ from the experimental data by measuring the squared statistical distance between probability distributions:

5 $${d}_{{\rm{H}}}^{\,2}({\theta }_{1},{\theta }_{2};N\,)=\mathop{\sum }\limits_{{J}_{z}=-N/2}^{N/2}\frac{1}{2}{\left(\sqrt{{p}_{{\theta }_{1}}(\,{J}_{z};N\,)}-\sqrt{{p}_{{\theta }_{2}}(\,{J}_{z};N)}\right)}^{2},$$

where *p*_*θ*_(*J*_*z*_; *N*) is the relative population of the output value *J*_*z*_ for a given rotation angle *θ*. A quadratic fit ${d}_{{\rm{H}}}^{2}({\theta }_{1},{\theta }_{2};N)=({F}_{N}({\theta }_{1})/8){({\theta }_{1}-{\theta }_{2})}^{2}$ provided an estimate of the Fisher information *F*_*N*_(*θ*_1_) (Methods). In the noise-free case, twin-Fock states allow reaching a Heisenberg scaling *F*_*N*_ = *N*^2^/2 + *N* with an improved robustness towards decoherence compared with the highest-sensitivity NOON states.

**Fig. 5:**
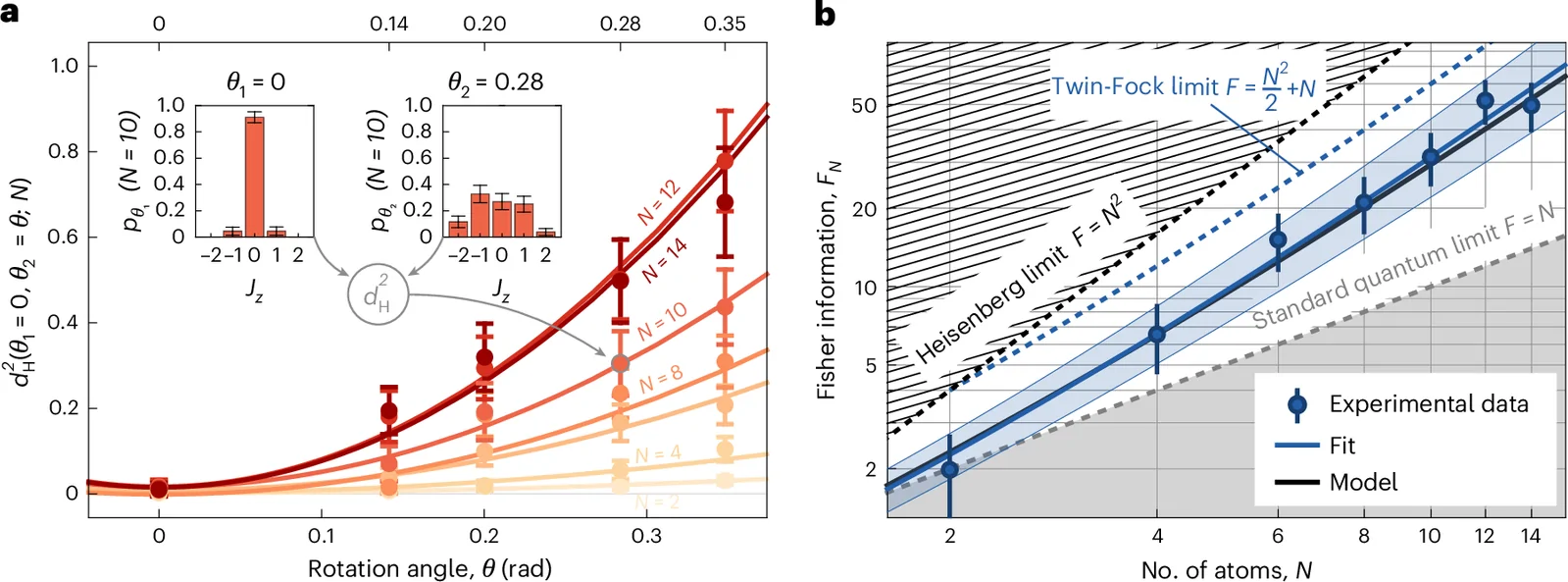
Heisenberg scaling of the Fisher information with the number of atoms. **a**, Insets: for each number of atoms *N* and rotation angle *θ*, the probabilities for different *J*_*z*_ values are estimated from the measured occurrences. The distinguishability of two probability distributions obtained at *θ*_1_ and *θ*_2_ is quantified by the squared Hellinger distance ${d}_{{\rm{H}}}^{2}({\theta }_{1},{\theta }_{2};N)$ (equation (5)). Main plot: the rate at which the Hellinger distance changes with the phase difference *θ*_1_ − *θ*_2_ provides information about the metrological sensitivity of the state. A quadratic fit $({F}_{N}({\theta }_{1})/8){({\theta }_{1}-{\theta }_{2})}^{2}$ to the calculated values of ${d}_{{\rm{H}}}^{2}$ allowed us to extract the Fisher information *F*_*N*_(*θ*_1_). **b**, The weighted average of *F*_*N*_(*θ*_1_) over different *θ*_1_ is shown as a function of *N*. The values obtained for the Fisher information increased with the number of atoms according to $r({N}^{s}/2+N)$. The demonstrated scaling exponent of *s* = 1.95(9) is compatible with the theoretical prediction for ideal twin-Fock states, *s* = 2, being a factor *r* = 0.57(10) below the ideal values. The blue shaded area depicts the 68% confidence region. The experimental data agree with a model that includes the combined effect of the dominant noise sources (Methods), with no free fitting parameters. The displayed mean values were derived from 391(21), 163(9), 96(14), 66(9), 49(4), 31(3) and 28(4) measured occurrences for numbers of atoms *N* = 2 to 14, respectively. The sample sizes deviated only slightly for the different rotation angles *θ*. For example, the insets in **a** show estimates obtained from 45 (*θ*_1_ = 0) and 52 (*θ*_1_ = 0.28) measured occurrences. The error bars in **a** were obtained as standard deviations from a Monte Carlo resampling approach (Methods), and those in **b** represent the averaged standard errors of the fitting parameters *F*_*N*_(*θ*_1_). Source data

We probed the metrological sensitivity by recording the relative population for four rotation angles close to *θ* = 0 with another 4 × 3,816 experimental realizations. The rotation angle quantifies the strength of the mode coupling. The insets of Fig. 5a show exemplary histograms obtained for a twin-Fock state with *N* = 10 atoms before and after rotation by *θ* = 0.28 rad (Extended Data Fig. 5). Although the input state has a negligible population of *J*_*z*_ ≠ 0, these contributions increased with rotation angle: an effect that was captured by the Fisher information. Figure 5a presents the squared Hellinger distance according to equation (5) between the twin-Fock state (*θ*_1_ = 0) and the rotated state for numbers of atoms ranging from *N* = 2 to 14, along with quadratic fits. Figure 5b shows the mean Fisher information ${\bar{F}}_{N}$ obtained by averaging the fitting results *F*_*N*_(*θ*_1_) over the available angles *θ*_1_. The obtained Fisher information significantly exceeded the classical limit *F*_*N*_ = *N* for unentangled states: for *N* = 12, we observed a Fisher information with a 6.4(8)-dB enhancement. Moreover, the Fisher information and the corresponding sensitivities increased with the same scaling obtainable in an ideal measurement using twin-Fock states. To quantify this observation, we modelled the data using the function ${\bar{F}}_{N}=r\,({N}^{s}/2+N)$, which is versatile enough to capture both the ideal twin-Fock scenario (where *s* = 2 and *r* = 1) and the classical limit (where *s* = 1 and r=2/3). A fit yielded ${\bar{F}}_{N}=0.57(10)\times ({N}^{1.95(9)}/2+N)$, which expresses the Heisenberg scaling of the interferometric sensitivity. Entanglement-enhanced sensitivities for neutral atoms have been demonstrated in thermal and condensed atomic ensembles^8^ with up to 20-dB squeezing enhancement^54^ for 10^6^ atoms. However, detection noise and other technical noise sources limited the achievable sensitivities to orders of magnitude above the Heisenberg limit. A Heisenberg scaling of the phase sensitivity has so far been demonstrated only with ions^48^.

## Discussion

The demonstrated HOM interference, including the generation and analysis of high-fidelity entangled many-particle states, opens a path towards quantum atom optics and atom interferometry with unprecedented fidelities, negligible losses and single-atom resolution. Our set-up and experimental capabilities allow for the further exploration of multipartite entanglement in complex many-body systems, like those generated by crossing quantum phase transitions. For instance, adiabatic passages enable the deterministic generation of twin-Fock states^40,55^ or even Schrödinger-cat-like states^56^. Furthermore, by realizing a spatial separation^57^, such states open the path to multiparticle Bell tests with both twin-Fock^58^ and two-mode squeezed states^59,60^. The proposal of ref. ^58^ requires small losses and the ability to measure parity, both of which we demonstrated with the presented results. Finally, the detection set-up can be improved to detect up to 1,000 atoms with single-atom resolution^44,61^. The state generation can be improved to the same number of atoms by reducing the density and the corresponding three-body collisions. Thus, our method promises to scale up the Heisenberg-limited sensitivities into an atom number regime, where the interferometric resolution becomes competitive with state-of-the-art unentangled sources, thus enabling the future generation of high-precision atom interferometers.

## Methods

### Coherent mode coupling and spin-changing collisions

We employed a low-noise 6.8-GHz microwave source^62^ to drive Rabi oscillations between the *F* = 1 and *F* = 2 manifolds. The transition frequencies were defined by an actively stabilized homogeneous magnetic field of 0.955 G. The microwave system was developed and tested for spin-squeezed samples of more than 10^4^ atoms in previous studies, where it contributed no relevant technical noise. Rabi pulse lengths are typically of the order of 100 μs. We implemented a spin-distillation scheme^63^ during the evaporative cooling, such that the BEC occupies the $\left(1,0\right)$ level, and the side modes $\left(1,\pm 1\right)$ are initially occupied by only a few atoms at most. Before the spin-changing collisions, we removed these atoms by transferring them to $\left(2,\pm 1\right)$ and exposing the ensemble to resonant cooling light. We repeated this cleaning sequence three times.

We aimed to prepare BECs with a small number of atoms of around 250 to avoid saturating the CCD camera during illumination and to reduce the effect of certain noise contributions, as discussed later. To maintain a high spin-dynamics rate *Ω* = 2π × 2.2 Hz, we increased the trap frequencies by raising the beam powers of the optical dipole trap from 23 mW and ~320 μW to 200 mW and 2 mW after BEC creation. The spin-dynamics rate scales as ${\it\varOmega} \propto {\bar{\omega }}^{6/5}{N}_{\mathrm{BEC}}^{2/5}$ with the geometric mean $\overline{\omega }$ of the trap frequencies and the numbers of atoms in the BEC *N*_BEC_. We applied a microwave dressing field on the clock transition to shift the Zeeman energy to resonance at *q* = *ℏΩ* (ref. ^43^), where *ℏ* is the reduced Planck constant. After 120 ms, we found a mean number of 7.5 atoms in the levels $\left(1,\pm 1\right)$. The distribution of the occupation numbers did not follow an exponential decay as predicted by equation (1). We ascribe this discrepancy to a non-constant spin-dynamics rate due to fluctuations in the numbers of atoms and trap frequencies.

To couple the levels $\left(1,\pm 1\right)$, we applied a sequence of three microwave Rabi pulses (Extended Data Fig. 1). Between two π-pulses, which transferred the atoms from $\left(1,-1\right)$ to $\left(2,0\right)$ and back, we used a pulse of variable length on $\left(2,0\right)\leftrightarrow \left(1,+1\right)$ to control the coupling ratio of the twin-Fock modes $\left(1,\pm 1\right)$. Effective pulse lengths of 7.66 μs, 10.8 μs, 15.4 μs, 18.8 μs and 85.0 μs at a Rabi frequency of ${\it\varOmega }_{{\rm{R}},{\sigma }^{-}}=2{\rm{\pi }}\times 2.94\,\mathrm{kHz}$ resulted in small rotation angles between 0.14 rad and 0.35 rad and the HOM coupling corresponding to an angle of π/2.

As the transition frequencies for $\left(1,0\right)\leftrightarrow \left(2,\pm 1\right)$ differed from those for $\left(1,\pm 1\right)\leftrightarrow \left(2,0\right)$ by only *Δ* = 2π × 2.66 kHz, the pulse sequence also transferred some BEC atoms to *F* = 2. These atoms were removed by a cooling light exposure during free fall, 2.5 ms before the detection beams were turned on. We observed that removing atoms this way can cause losses of $\left(1,\pm 1\right)$ atoms. We, thus, kept the fraction of BEC atoms transferred to *F* = 2 small by using two different techniques. For the coupling pulse, we chose a microwave antenna that couples 4.7 times less to *σ*^+^ than to *σ*^−^ transitions, that is ${\it\varOmega }_{{\rm{R}},{\sigma }^{+}}={\it\varOmega }_{{\rm{R}},{\sigma }^{-}}/4.7$, resulting in a maximally transferred fraction of ${\varOmega }_{{\rm{R}},{\sigma }^{+}}^{2}/({\varOmega }_{{\rm{R}},{\sigma }^{+}}^{2}+{\varDelta }^{2})\approx 2 \%$. For the two π-pulses, we chose the relative phase such that all BEC atoms that were transferred by the first pulse were transferred back to $\left(1,0\right)$ by the second pulse.

### Fluorescence detection

For detection, the atomic ensemble was released from the crossed-beam optical dipole trap, exposed to a magnetic field gradient pulse during free fall and then illuminated by six laser beams in an optical-molasses configuration (Extended Data Fig. 2a). The magnetic field gradient was generated by a single coil aligned with the homogeneous magnetic quantization field used for the coherent state manipulation. Then, 0.5 ms after release, a capacitor was discharged through the coil over a period of 6 ms, resulting in a peak current of 430 A and a gradient of about 40 G cm^−1^ at the position of the atoms. A spatial separation of 470 μm was reached for adjacent modes. At the end of the pulse, 6.5 ms into free fall, both magnetic fields were turned off. After another 3.5 ms, during which time the fields settled down, the laser beams of the optical molasses were turned on.

The molasses consisted of six millimetre-sized, circularly polarized beams, detuned by one natural linewidth *Γ* to the $F=2\to {F}^{{\prime} }=3$ cooling transition of the ^87^Rb D_2_ line 5^2^S_1/2_ → 5^2^P_3/2_. The optical set-up was the same as that described in ref. ^46^, but with the beam diameters of the four horizontal beams reduced from 3.1 mm to 1.1 mm. This adaptation reduced the amount of stray light present during the illumination process, which was a main contributor to the counting noise. Beam intensities close to the saturation intensity for isotropically polarized light, *I*_sat_ = 3.6 mW cm^−^^2^, resulted in an expected isotropic photon scattering rate of *R* = 1.04 × 10^7^ photons per second. Considering the photon collection efficiency of the detection lens system of 3.9% and the properties of the CCD camera employed (Pixis 1024BR eXcelon WaterCool, 1,024 × 1,024), which are given by a quantum efficiency of 0.98 primary electrons per incident photon and a set amplification gain of 0.92 digital counts per electron, we expected 1,530 counts during the illumination time of 4.2 ms per atom. From the recorded data, we found 978 counts per atom in $\left(1,-1\right)$, 1,580 counts per atom in $\left(1,0\right)$ and 830 counts per atom in $\left(1,+1\right)$. As the molasses beams were aligned onto the position of the $\left(1,0\right)$ atoms after 10 ms of free fall, these atoms experience the most intense and best intensity-balanced light field and, thus, emitted the most fluorescence photons. The twin-Fock modes probably experienced a slight intensity-imbalance of counter-propagating beams, as the horizontal displacement of 470 μm was not negligible compared with the Gaussian beam waists of about 550 μm.

### Image evaluation

The CCD camera array has 1,024 × 1,024 physical pixels. Incident photons lead to the accumulation of charge during the illumination time. To reduce the level of electronic noise, arrays of 8 × 8 were combined into a super-pixel (px in the following) before read-out, resulting in an image of size 128 px × 128 px. For the three modes $\left(1,-1\right)$, $\left(1,0\right)$ and $\left(1,+1\right)$, we added up the brightness values of the pixels in constant areas (‘masks’) around the respective bright spot so that we had a single camera-count value for each image and mode, denoted *s*_0_ and *s*_±_ for *m*_*F*_ = 0 and *m*_*F*_ = ± 1, respectively. We found the best results for round areas with radii of 5 px. Each mask had 69 (super-)pixels, with one (super-)pixel covering a physical area of 34.4 μm × 34.4 μm at the focus plane of the detection objective. We took one image for each run of the experimental apparatus. No background image was taken.

**Extended Data Fig. 3:**
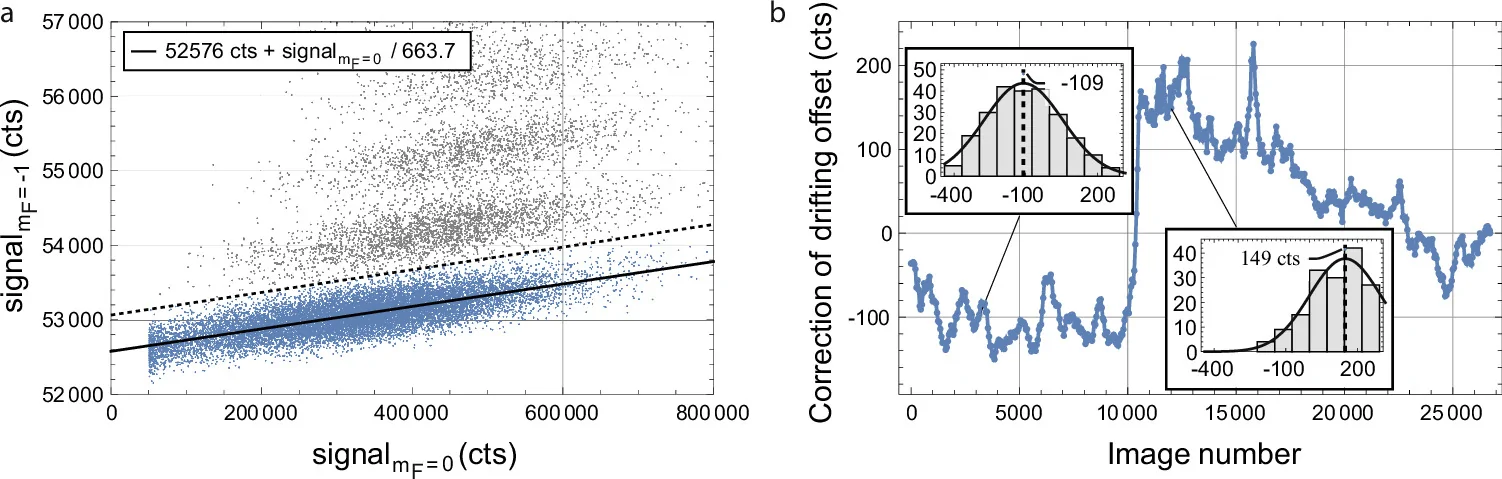
Camera signal corrections. **a**, Due to movement of *m*_*F*_ = 0 atoms during the illumination, a small fraction of their fluorescence signal is present in the neighboring masks. The correlation is captured with a linear fit (solid black line) to the signals of zero *m*_*F*_ = − 1 atoms (blue points), and subsequently subtracted. **b**, A small drift of the background light intensity can be extracted from the recorded camera signals. For this, a single Gaussian function is fitted to the zero-atom signal peak of the histogram of 400 adjacent images. Subsequently, the peak position is subtracted from those images recorded at similar times. The data presented here is for the *m*_*F*_ = − 1 mode. The data for the *m*_*F*_ = + 1 mode looks similar. Source data

A quantization of the signals, that is an accumulation of the obtained count numbers at evenly spaced values, was visible in the raw data. Importantly, the mean signals of the zero-atom peaks were easily evaluated by a fit with a single Gaussian. To obtain the data shown in Fig. 2b, we calculated and subtracted the contributions of two systematic effects (Extended Data Fig. 3). First, a very small but still relevant fraction of the *m*_*F*_ = 0 atoms moved into the regions of the *m*_*F*_ = ±1 atoms during illumination, which caused correlations of the zero-atom signals ${s}_{\pm }^{({N}_{\pm }=0)}$ and the $\left(1,0\right)$ signal *s*_0_, with correlation coefficients 1.48 × 10^−3^ (*m*_*F*_ = −1) and 1.76 × 10^−3^ (*m*_*F*_ = +1). Second, we found that the zero-atom signals ${s}_{\pm }^{({N}_{\pm }=0)}$, identified from 400 adjacent images, drifted slightly over the duration of the measurement, with the maximal and minimal observed signals differing by 370 counts.

Finally, the occurrences of the count values were fitted with a sum of evenly spaced Gaussian functions:

6 $$G(s)=\mathop{\sum }\limits_{n=0}^{{n}_{\max }+1}{a}_{n}\,\exp \left(\frac{{(s-(ng+b))}^{2}}{2{\sigma }_{n}^{2}}\right),$$

where *a*_*n*_ are the peak heights, *σ*_*n*_ the peak widths and *g* the signal per atom. The position of the zero-atom peak *b* was close to zero due to the applied signal drift correction. For quantization, each camera-count value per image and mode was assigned the integer number of atoms *n* of the closest peak, resulting in the quantization intervals depicted in Fig. 2b and Extended Data Fig. 2b, respectively. Note that this quantization technique extends to numbers of atoms much larger than ${n}_{\max }$, the number for the last peak that could be fitted.

For the fitting procedure, we weighted the occurrences within each peak with the inverse of the total number of the detection events for the peak. To ensure convergence, the ${n}_{\max }+1$ peak was assigned a fixed width (which we predicted iteratively from the widths of the previous peaks). The fits with equation (6) yielded atomic fluorescence signals *g* of 832.5(34) counts per atom for the *m*_*F*_ = +1 mode and 975.8(16) counts per atom for the *m*_*F*_ = −1 mode. The counting noise was captured in the widths *σ*_*n*_. A noise model of the molasses detection predicted electronic camera noise and background light fluctuations (from shot noise and non-constant beam powers) to be the dominant contributors to the zero-atom signal noise, denoted *σ*_0_. The most relevant contribution that scales with the number of atoms was from atoms leaving the detection volume during the illumination time due to the slowed, but not spatially restricted, atom movement in the optical molasses. This is captured by *c*_1_ (ref. ^44^), and we get

7 $${\sigma }_{n}^{2}={\sigma }_{0}^{2}+{c}_{1}^{2}n.$$

The fits with equation (7) to the obtained Gaussian widths of the peaks are presented in Fig. 2c and Extended Data Fig. 2c. For the *m*_*F*_ = −1 mode, we found *σ*_0_ = 0.1466(9) atoms, incoherently increasing by ${c}_{1}=0.107(3)\,{\rm{atoms}}/\sqrt{\,{\rm{atoms}}}$, whereas for *m*_*F*_ = +1, we found *σ*_0_ = 0.168(4) atoms and ${c}_{1}=0.164(15)\,\,{\rm{atoms}}/\sqrt{{\rm{atoms}}}$. From this, we estimated the detection fidelities, that is the chance of correctly counting the number of atoms, as the integral of the normalized Gaussian functions over the respective quantization intervals. For up to *N* = 12 atoms, we obtained fidelities of >79% for the *m*_*F*_ = −1 mode and >60% for the *m*_*F*_ = +1 mode. The larger coefficient *c*_1_ for *m*_*F*_ = +1 can be explained by a less optimal beam intensity balance at the position of these atoms after the spatial separation.

### Calculating fidelities ${\mathcal{F}}$

For two probability distributions *p*(*J*_*z*_) and *q*(*J*_*z*_), the fidelity is given as ${\mathcal{F}}={\left({\sum }_{{J}_{z}}\sqrt{p({J}_{z})q({J}_{z})}\right)}^{2}$. For the numbers given in Fig. 3, we compared the experimentally observed probabilities ${p}^{\exp }({J}_{z};N)$ with the probability distributions *q*(*J*_*z*_) of the ideal quantum states, where *J*_*z*_ = −*N*/2, −*N*/2 + 1, …, *N*/2 represents all possible values for a given even number of atoms *N*. The experimental probabilities ${p}^{\exp }({J}_{z};N)$ were obtained from the number of occurrences of the value *J*_*z*_ during the full measurement. The probability distributions for the ideal states are given by $q({J}_{z})={\delta }_{0,{J}_{z}}$ for the twin-Fock states, where ${\delta }_{0,{J}_{z}}$ is the Kronecker delta, and by equation (3) for the states after HOM interference. Expressed in terms of *J*_*z*_ and *N*:

$$q(\,{J}_{z})=\left\{\begin{array}{cc}\left(\begin{array}{l}N/2+{J}_{z}\\ (N/2+{J}_{z})/2\end{array}\right)\left(\begin{array}{l}N/2-{J}_{z}\\ (N/2-{J}_{z})/2\end{array}\right){\left(\frac{1}{2}\right)}^{N}, & {J}_{z}+N/2\,\mathrm{even},\\ 0, & {J}_{z}+N/2\,\mathrm{odd}.\end{array}\right.\,$$

### Parity

The parity operator for a single mode assigns a value of +1 to even occupation numbers and −1 to odd occupation numbers^64^. As all states can be written as a superposition of Fock states $\left|n\right\rangle$, this property fully defines the operator. It can be written as ${\it\varPi }_{\mathrm{single}\,\mathrm{mode}}={(-1)}^{\widehat{n}}$, with $\widehat{n}=\left|n\right\rangle \left\langle n\right|$ the occupation number operator.

For our two-mode system, in principle, two parity operators exist, ${\it\varPi }_{+}={(-1)}^{{\widehat{N}}_{+}}$ and ${\it\varPi }_{-}={(-1)}^{{\widehat{N}}_{-}}$. However, for an even total number of atoms *N* = *N*_+_ + *N*_−_, we note that (−1)^*N*^ ≡ 1, such that

$${\it\varPi }_{z\,}:={(-1)}^{N/2-{J}_{z}}={(-1)}^{{N}_{-}}={(-1)}^{{N}_{+}}$$

is well defined and describes the occupation number parity for both of the measured modes. Similarly, we can define parity operators for all spin components *J*_*l*_, with *l* = *x*, *y* and *z*, as

$${\it\varPi }_{l\,}:={(-1)}^{N/2-{J}_{l}}.$$

*Π*_*x*,*y*_ describe parity measurements after rotating the state by 90° on the generalized Bloch sphere, that is after HOM interference. Their relation to the single-particle operators is ${\it\varPi }_{l}={\sigma }_{l}^{\otimes N}$, with *σ*_*l*_ = 2*j*_*l*_ twice the *l* component of the single-particle spin-1/2 operator, which can be written as a Pauli matrix and has eigenvalues of ±1.

### Probabilistic noise model of the measurements

We deliberately avoid using assumptions about the underlying noise sources in the analysis of the recorded population data. In the absence of any noise correction technique, the results presented directly describe the performance of our system.

To compare the results with theoretical expectations and for consistency checks, we developed a numerical model that describes the combined effect of various noise contributions on the population probabilities *p*_*θ*_(*J*_*z*_; *N*). These probabilities for the possible outcomes (*J*_*z*_; *N*) are modelled as an array, ${p}_{\theta }^{{\rm{model}}}({J}_{z};N)$, like those depicted in the insets of Fig. 2d,e. To ensure that truncation effects were negligible, the model considered up to *N* = 20 atoms per mode, which is a range much larger than that used for Figs. 3–5. We checked that the results were stable with respect to changes in the array size. The calculation of the probabilities follows these steps:

1. Start with the probabilities of a superposition of twin-Fock states. The distribution of the total numbers of atoms *N* follows the recorded average of all measurements.
2. Change the probabilities within the subspaces of constant *N* according to a rotation by the angle *θ*. For example, *θ* = 90° results in Holland–Burnett states.
3. Undesired transfers of atoms in the BEC reservoir into the detected modes *m*_*F*_ = ±1 can occur after the mode interference when the magnetic fields change quickly before and during the strong magnetic field gradient pulse for spatial separation.
  These extra particles are modelled by a convolution of the probability array with Poisson distributions with parameters *a*_±_.
4. When those BEC atoms that were transferred to $\left(F=2,{m}_{F}=\pm 1\right)$ during the Rabi coupling sequence (Extended Data Fig. 1) are removed by a short resonant light pulse after the magnetic field gradient, losses in $\left(F=1,{m}_{F}=\pm 1\right)$ can occur due to collisions with the accelerated atoms. This effect is clearly visible when we purposely remove very many atoms.
  These losses are described by convolutions with binomial distributions with probabilities 1 − *l*_±_.
5. The calibration of the detection, that is the definition of the quantization intervals shown in Fig. 2b and Extended Data Fig. 2b, will have some error. Looking at measurement outcomes for twin-Fock states with very high numbers of atoms *N*, we notice that the detection predicts slightly asymmetric numbers *N*_−_ ≈ 1.052*N*_+_.
  This is modelled by applying chances of $\sqrt{1.052}-1$ per atom to overpredict *N*_−_ by 1 and underpredict *N*_+_ by 1. Note that we cannot use this observation to refine the assignment of the number of atoms when analysing the experimental data. That calibration method would assume, rather than demonstrate, the generation of twin-Fock states.
6. Finally, the finite detection resolution, that is counting noise, is considered by miscounting probabilities according to the overlap of the Gaussian peaks from equation (6) with adjacent quantization intervals.

The model has only four free parameters, namely *a*_±_ and *l*_±_.

For each value of the rotation angle *θ* (0 rad, 0.14 rad, 0.20 rad, 0.28 rad and 0.35 rad), we fitted the model to the full array of measured experimental probabilities ${p}_{\theta }^{\exp }({J}_{z};N)$ for 0 ≤ *N*_±_ ≤ 20, normalized such that

8 $$\mathop{\sum }\limits_{N=0}^{20}\mathop{\sum }\limits_{{J}_{z}=-N/2}^{N/2}{p}_{\theta }^{\exp }(\,{J}_{z};N\,)=1.$$

Note that this analysis includes odd values of *N*, which are the primary effect of the noise contributions. For the fitting, we minimized the Hellinger distance between ${p}_{\theta }^{{\rm{model}}}({J}_{z};N)$ and ${p}_{\theta }^{\exp }({J}_{z};N)$ using a differential evolution algorithm.

For each *θ*, we performed 3,816 experimental repetitions, each resulting in a pair (*N*_+_, *N*_−_) of detected atoms. We obtained fitting parameters *a*_+_ = 0.0551(63), *a*_−_ = 0.0218(18), *l*_+_ = 0.042(22)% and *l*_−_ = 1.1(8)% as the mean values for the five angles *θ*. The uncertainties are the statistical standard deviation. We attribute the increased chance for losses in *m*_*F*_ = −1 to the asymmetry of the coupling sequence, as a small fraction of atoms might not be transferred back to $\left(F=1,{m}_{F}=-1\right)$ by the second π-pulse due to magnetic field fluctuations.

The obtained parameters indicate that losses have only a minor impact on our system. The primary noise sources were finite detection resolutions, as depicted in Fig. 2c and Extended Data Fig. 2c, as well as unintended incoherent transfers from $\left(F=1,{m}_{F}=0\right)$ to $\left(F=1,{m}_{F}=\pm 1\right)$, as described by *a*_±_.

For clarity, note that the results of the noise model were not incorporated in the data analysis for Figs. 3–5.

### Entanglement witness based on parity

We considered the detection of entanglement in systems with many indistinguishable particles, using the definition of particle entanglement as described, for example, in ref. ^6^. The first important result in this field was the spin-squeezing entanglement condition based on the first and second moments of collective observables^5^. Subsequently, many experiments measured collective quantities^40,50,65–67^.

In this section, we present entanglement relations that are based on *N*-particle correlations, rather than the first and second moments of collective observables.

We can use the following witness to detect entanglement. For separable states

9 $$| \langle {\it\varPi }_{x}\rangle | +| \langle {\it\varPi }_{y}\rangle | +| \langle {\it\varPi }_{z}\rangle | \le 1$$

holds, which can be proved following ideas like those in ref. ^68^. For a product state of the type

10 $$\left|{\varPsi }^{(1)}\right\rangle \otimes \left|{\varPsi }^{(2)}\right\rangle \otimes \ldots \otimes \left|{\varPsi }^{(N)}\right\rangle ,$$

the left-hand side of equation (9) can be bounded from above as

11 $$\mathop{\sum }\limits_{l=x,y,z}\left|\mathop{\prod }\limits_{n=1}^{N}\left\langle {\sigma }_{l}^{(n)}\right\rangle \right|\le \mathop{\sum }\limits_{l=x,y,z}\left|\left\langle {\sigma }_{l}^{(1)}\right\rangle \left\langle {\sigma }_{l}^{(2)}\right\rangle \right|\le 1,$$

where in the first inequality we used that $| \langle {\sigma }_{l}^{(n)}\rangle | \le 1$. In the second inequality, we used the Cauchy–Schwarz inequality and the fact that the length of the Bloch vector is at most 1 for a qubit. Separable states are mixtures of product states. Hence, the inequality in equation (9) is also valid for separable states.

For the ideal Dicke state, for even *N*, the left-hand side is 3. This condition is based on *N*-body correlations, unlike previous methods that were based on two-body correlations. Here *Π*_*l*_ is the parity operator, which equals ${\sigma }_{l}^{\otimes N}$ for *l* = *x*, *y* and *z*.

The witness also detects the Greenberger–Horne–Zeilinger states as entangled. The singlet state, given as ${[(\left|01\right\rangle -\left|10\right\rangle )/\sqrt{2}]}^{\otimes N/2}$, has ${(\Delta {J}_{z})}^{2}=0$ and also $\langle {\sigma }_{x}^{\otimes N}\rangle =1$ and $\langle {\sigma }_{y}^{\otimes N}\rangle =1$, if *N* is divisible by 4. Thus, these operators cannot be used to detect genuine multipartite entanglement.

We summarize the results in Extended Data Table 1. We assume $\langle {\sigma }_{y}^{\otimes N}\rangle =\langle {\sigma }_{x}^{\otimes N}\rangle .$ The witness detects entanglement in all cases.

Next, we will derive an entanglement condition detecting genuine multipartite entanglement based on the parity operator *Π*_*z*_. As a first step, we will derive a criterion that detects entanglement between two groups of the particles.

### Entanglement condition using bipartite correlations

In this section, we present a simple relation using the expectation values of collective observables and the expectation values of *N*-particle correlations. We use these relations to obtain entanglement conditions based on bipartite correlations that detect entanglement between two groups of particles.

#### Observation 1

For *N*-qubit quantum states,

12 $${\langle \,{J}_{x}\rangle }^{2}/{j}^{2}+{\langle \,{J}_{y}\rangle }^{2}/{j}^{2}+{\langle {\sigma }_{z}^{\otimes N}\rangle }^{2}\le 1$$

holds, where *j* = *N*/2 and

13 $${J}_{l}=\frac{1}{2}\mathop{\sum }\limits_{n=1}^{N}{\sigma }_{l}^{(n)}$$

for *l* = *x*, *y*, *z*.

*Proof*. The ground state of the Hamiltonian $H=B{J}_{x}+K{\sigma }_{z}^{\otimes N},$ where *B* and *K* are constants, is of the form $\left|\Psi \right\rangle =\alpha {\left|0\right\rangle }_{x}^{\otimes N}+\beta {\left|1\right\rangle }_{x}^{\otimes N},$ which is a generalized Greenberger–Horne–Zeilinger state in the *x* basis. Then, the relevant expectation value of *J*_*x*_ is $\langle {J}_{x}\rangle =\frac{N}{2}{\langle {\sigma }_{x}\rangle }_{\phi }$ and the expectation value of the products of *σ*_*z*_ matrices is $\langle {\sigma }_{z}^{\otimes N}\rangle ={\langle {\sigma }_{z}\rangle }_{\phi },$ where we define the single-qubit state $\left|\phi \right\rangle =\alpha {\left|0\right\rangle }_{x}+\beta {\left|1\right\rangle }_{x}.$ As ${\langle {\sigma }_{x}\rangle }_{\phi }^{2}+{\langle {\sigma }_{z}\rangle }_{\phi }^{2}\le 1,$ it follows that ${\langle {J}_{x}\rangle }^{2}/{j}^{2}+{\langle {\sigma }_{z}^{\otimes N}\rangle }^{2}\le 1.$ Then, assuming that the mean spin is not in the *x* direction but is in the *x–y* plane, we arrive at equation (12).

#### Observation 2

For bipartite separable states,

14 $$\langle \,{J}_{x}\otimes {J}_{x}\rangle /(\,{j}_{1}\,{j}_{2})+\langle \,{J}_{y}\otimes {J}_{y}\rangle /(\,{j}_{1}\,{j}_{2})+\left|\begin{array}{c}\left\langle {\sigma }_{z}^{\otimes {N}_{1}}\otimes {\sigma }_{z}^{\otimes {N}_{2}}\right\rangle \end{array}\right|\le 1$$

holds, where *j*_1_ = *N*_1_/2 and *j*_2_ = *N*_2_/2.

*Proof*. We start from equation (12) and use the Cauchy–Schwarz inequality. See, for example, ref. ^68^.

### Entanglement-depth condition based on parity

In this section, we obtain entanglement criteria for detecting entanglement between two groups of particles. We start from the relations based on bipartite correlations. Then, we obtain entanglement conditions that do not need bipartite correlations but rather need the measurement of collective quantities. Such quantities can be measured even in systems in which we cannot address the particles individually. Finally, we present a relation that detects the entanglement depth and can detect genuine multipartite entanglement.

#### Observation 3

The following expression is true for bipartite separable states:

15 $$\mathop{\sum }\limits_{l=x,y}\left\langle {\left(\,{J}_{l}^{\,(1)}+{J}_{l}^{\,(2)}\right)}^{2}\right\rangle /(2{j}_{1}\,{j}_{2})+\left|\left\langle {\sigma }_{z}^{\otimes N}\right\rangle \right|\le j(j+1)/(2{j}_{1}\,{j}_{2}),$$

where *j*_1_ = *N*_1_/2, *j*_2_ = *N*_2_/2 and *j* = *N*/2.

*Proof*. We start from equation (14). We add to both sides ${\sum }_{l=x,y}\langle {(\,{J}_{l}^{(1)})}^{2}\rangle /(2{j}_{1\,}{j}_{2})+\langle {(\,{J}_{l}^{(2)})}^{2}\rangle /(2{j}_{1}{j}_{2})$ to give:

16 $$\mathop{\sum }\limits_{l=x,y}\left\langle {\left(\,{J}_{l}^{(1)}+{J}_{l}^{\,(2)}\right)}^{2}\right\rangle /(2{j}_{1}\,{j}_{2})+\left|\left\langle {\sigma }_{z}^{\otimes N}\right\rangle \right|$$

17 $$\le 1+\mathop{\sum }\limits_{l=x,y}\left\langle {\left(\,{J}_{l}^{(1)}\right)}^{2}\right\rangle /(2{j}_{1}\,{j}_{2})+\left\langle {\left(\,{J}_{l}^{(2)}\right)}^{2}\right\rangle /(2{j}_{1}\,{j}_{2}).$$

Finally, we use the inequality $\langle {({J}_{x}^{(n)})}^{2}+{({J}_{y}^{(n)})}^{2}\rangle \le {j}_{n}({j}_{n}+1).$

Next, we will show how to use the criterion given in equation (15) to detect genuine multipartite entanglement of the *N*-qubit system.

#### Observation 4

States violating the inequality given in equation (15) for *j*_1_ = *k*/2 and *j*_2_ = (*N* − *k*)/2 have at least (*k* + 1)-particle entanglement, where we assume that *k* ≥ *N*/2. A violation for *k* = *N* −1 means genuine multipartite entanglement (equation (4)).

*Proof*. The violation of equation (15) for *j*_1_ = *k*/2 means that the state cannot be written as a mixture of states of the form

18 $$\left|{\varPsi }_{1}\right\rangle \otimes \left|{\varPsi }_{2}\right\rangle ,$$

where $\left|{\varPsi }_{1}\right\rangle$ has *k* qubits and $\left|{\varPsi }_{2}\right\rangle$ has (*N* − *k*) qubits. Note that this is true for any separation of the qubits into groups of *k* and (*N* − *k*) qubits. The states given in equation (18) are called biseparable states, as they are possibly multipartite entangled states that are separable with respect to a bipartition.

Without loss of generality, let us consider the case $\langle {\sigma }_{z}^{\otimes N}\rangle \ge 0$. Then, let us rewrite the inequality given in equation (15) as

19 $$\mathop{\sum }\limits_{l=x,y}\left\langle {\left(\,{J}_{l}^{\,(1)}+{J}_{l}^{(2)}\right)}^{2}\right\rangle +2{j}_{1}\,{j}_{2}\left\langle {\sigma }_{z}^{\otimes N}\right\rangle \le j(\,j+1).$$

The product *j*_1_ *j*_2_ = *j*_1_(*j* − *j*_1_) is largest for *j*_1_ = *j*/2, and it is monotonically decreasing for a decreasing *j*_1_. It is also monotonously decreasing if *j*_1_ is increasing from *j*_1_ = *j*/2. It is the smallest for *j*_1_ = 1/2 and *j*_2_ = *N*/2 − 1/2 and for *j*_2_ = 1/2 and *j*_1_ = *N*/2 − 1/2. In general, the value of *j*_1_(*j* − *j*_1_) is the same for *j*_1_ as for *j* − *j*_1_. Hence, if the criterion in equation (19) is violated by a quantum state for *j*_1_ ≤ *j*/2, then it is also violated for any ${j}_{1}^{{\prime} }$ fulfilling ${j}_{1}\le {j}_{1}^{{\prime} }\le j-{j}_{1}.$

Let us now consider the criterion in equation (19) with *j*_1_ = *k*/2, where we assumed that *k* ≥ *N*/2. Let us consider pure biseparable states of the form equation (18), such that $\left|{\varPsi }_{1}\right\rangle$ has *N*_*Ψ*1_ qubits and $\left|{\varPsi }_{2}\right\rangle$ has *N*_*Ψ*2_ qubits, and *N*_*Ψ*1_ ≥ *N*_*Ψ*2_, which also implies *N*_*Ψ*1_ ≥ *N*/2. Such a state can contain at most *N*_*Ψ*1_-particle entanglement. Then, pure biseparable states of the form of equation (18) with *N*_*Ψ*1_ ≤ *k* cannot violate the criterion. Moreover, as equation (19) is linear in the expectation values, such a criterion cannot be violated, even by states that are the mixtures of pure states of the type given in equation (18), $\left|{\varPsi }_{1}\right\rangle$ having *k* or fewer qubits. Simple arguments then show that the criterion in equation (19) cannot be violated by *k*-producible states, that is, states with at most *k*-particle entanglement. Thus, a state violating the criterion must have at least (*k* + 1)-particle entanglement or, equivalently, it must have at least an entanglement depth of (*k* + 1). It can be proven that states with at most *l*-particle entanglement with *l* < *N*/2 cannot violate the main condition given in equation (4) for any *k*.

If a state violates the criterion given in equation (19) for *j*_1_ = 1/2, then such a state cannot be a mixture of biseparable states of the form of equation (18) with $\left|{\varPsi }_{1}\right\rangle$ and $\left|{\varPsi }_{2}\right\rangle$ being quantum states of one or more qubits. Thus, the quantum state must be genuine multipartite entangled.

Observation 4 can be used to detect (*k* + 1)-particle entanglement such that *k* ≥ *N*/2. The expectation values used for the entanglement criterion are shown in Extended Data Table 1. In the table, we also give the value of the parameter

20 $${\mathcal{J}}=\frac{\left\langle \,{J}_{x}^{2}+{J}_{y}^{2}\right\rangle }{N(N+2)/4},$$

which characterizes how symmetric the state is. ${\mathcal{J}}=1$ corresponds to perfect bosonic symmetry.

### Entanglement-depth condition based on collective measurements of spin components

Let us use the definitions of the angular momentum components

21 $${J}_{l}=\frac{1}{2}\mathop{\sum }\limits_{n=1}^{N}{\sigma }_{l}^{(n)}$$

for *l* = *x*, *y* and *z*. An entanglement condition is defined in ref. ^50^. We use a somewhat stronger inequality in ref. ^52^, such that for states with at most *k*-particle entanglement, the following inequality holds

22 $${(\Delta {J}_{z})}^{2}\ge {J}_{\max }{F}_{k/2}\left(\sqrt{\frac{\left\langle \,{J}_{x}^{2}+{J}_{y}^{2}\right\rangle -{J}_{\max }(k/2+1)}{{J}_{\max }(\,{J}_{\max }-k/2)}}\right),$$

where the maximal spin length is defined as ${J}_{\max }=N/2,\,{F}_{j}(.)$ is defined in ref. ^69^. If the above condition is violated, we have at least (*k* + 1)-particle entanglement.

The criterion in ref. ^50^ can be improved in a different way in certain cases. We can detect (*k* + 1)-particle entanglement with a new condition:

23 $${(\Delta {J}_{z})}^{2}\ge {J}_{\max }{F}_{k/2}\left(\frac{\sqrt{\left\langle \,{J}_{x}^{2}+{J}_{y}^{2}\right\rangle -X}}{{J}_{\max }}\right).$$

Here we used that the maximum of ${(\Delta {J}_{x})}^{2}+{(\Delta {J}_{y})}^{2}$ for pure states that are at most *k*-particle entangled is^70,71^

24 $$X=\lfloor N/k\rfloor R(k)+R(r),$$

where we define

25 $$R(n)=\left\{\begin{array}{ll}n/2(n/2+1), & \,{\rm{even}}\,n,\\ n/2(n/2+1)-1/4, & \,{\rm{odd}}\,n.\end{array}\right.$$

For an *n*-qubit state, ${(\Delta {J}_{x})}^{2}+{(\Delta {J}_{y})}^{2}\le R(n)$ holds. Moreover,

26 $$r=N-\lfloor N/k\rfloor k.$$

**Extended Data Fig. 4:**
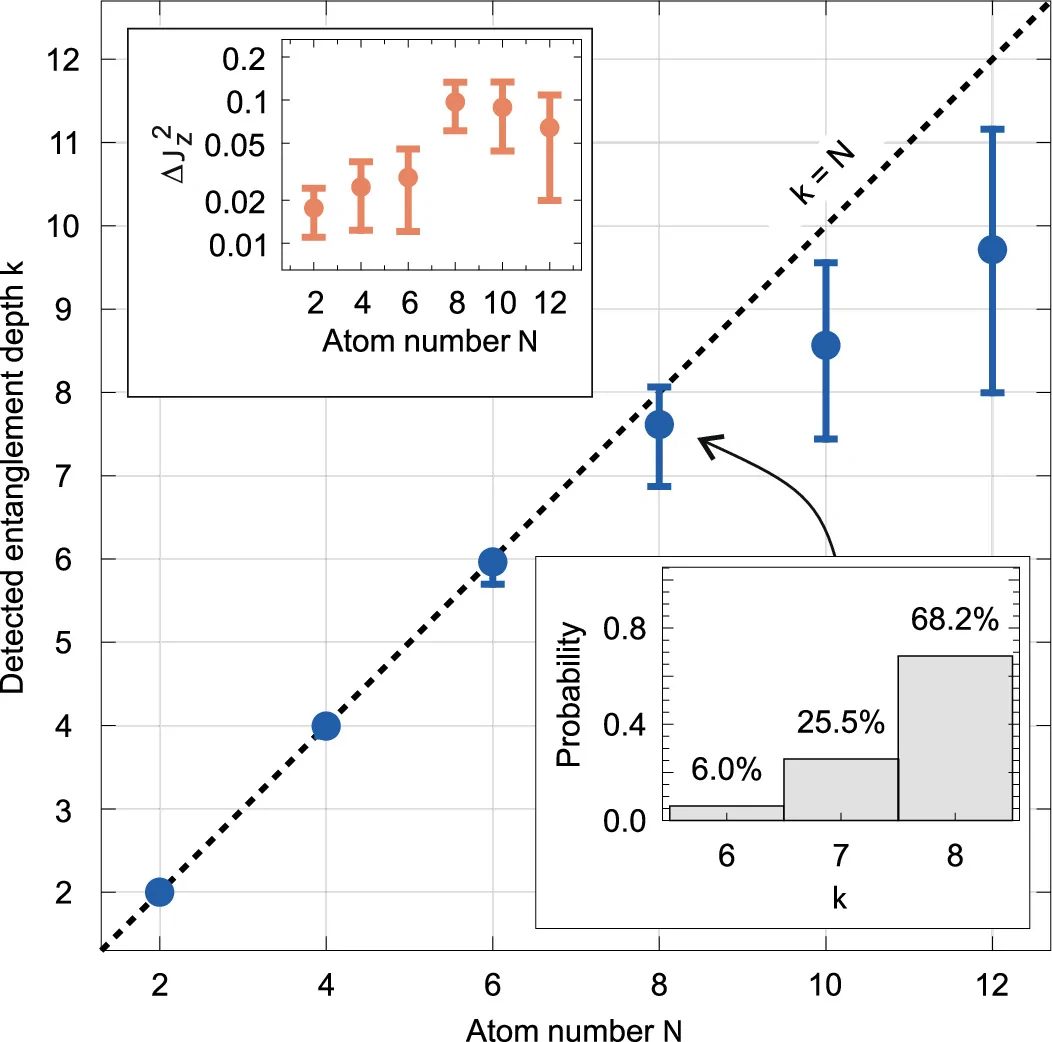
Multi-particle entanglement. The detected entanglement depth with the method based on variance ${(\Delta {J}_{z})}^{2}$. The criteria are given in equation (22) and equation (23). The error bars represent asymmetric standard deviations calculated via the Monte Carlo resampling approach described in section *Error calculation*. (Top left inset) The values of ${(\Delta {J}_{z})}^{2}$ for various *N*. The error bars denote the standard error of the mean. (Bottom right inset) The resampling approach results in a discrete distribution of *k* values, shown here for *N* = 8 as an example. The data in the figure represent mean values derived from the counting statistics shown in Fig. 3a,b. Source data

We used both conditions on the experimental data. For *k* = 1, we used the condition for entanglement in ref. ^72^. We chose the results from the method that gave a larger entanglement depth. The results are shown in Extended Data Fig. 4, and the expectation values used for the criterion are given in Extended Data Table 1.

Note that for states in the *J*_*z*_ = 0 subspace, 〈*J*_*x*_〉 = 〈*J*_*y*_〉 = 0, and for states with at most *k*-particle entanglement:

27 $$\left\langle \,{J}_{x}^{2}+{J}_{y}^{2}\right\rangle \le \lfloor N/k\rfloor R(k)+R(r)$$

holds. Any state that violates equation (27) is at least (*k* + 1)-particle entangled.

### Entanglement witness for a state with an indefinite number of particles

For separable states with a given number of particles, we have^72^

28 $$(N-1){(\Delta {J}_{z})}^{2}-\left\langle \,{J}_{x}^{2}+{J}_{y}^{2}\right\rangle +\frac{N}{2}\ge 0.$$

Note that the left-hand side is identical to zero for *N* = 0 and *N* = 1.

Then, if the number of particles is fluctuating, we use^73^

29 $$\begin{array}{rcl}{(\Delta {J}_{z})}^{2} & - & \left\langle {(N-1)}^{-1}{J}_{x}^{2}\right\rangle -\left\langle {(N-1)}^{-1}{J}_{y}^{2}\right\rangle \\ & + & \frac{1}{2}\left\langle {(N-1)}^{-1}N\right\rangle \ge 0,\end{array}$$

for which the left-hand side is −1.205(44), more than 27 standard deviations below zero. To compute the expectation values, we used the data for *N* = 2, 3, 4, …, 12 particles, which includes odd numbers of particles.

### Extracting the Fisher information from the Hellinger distance

**Extended Data Fig. 5:**
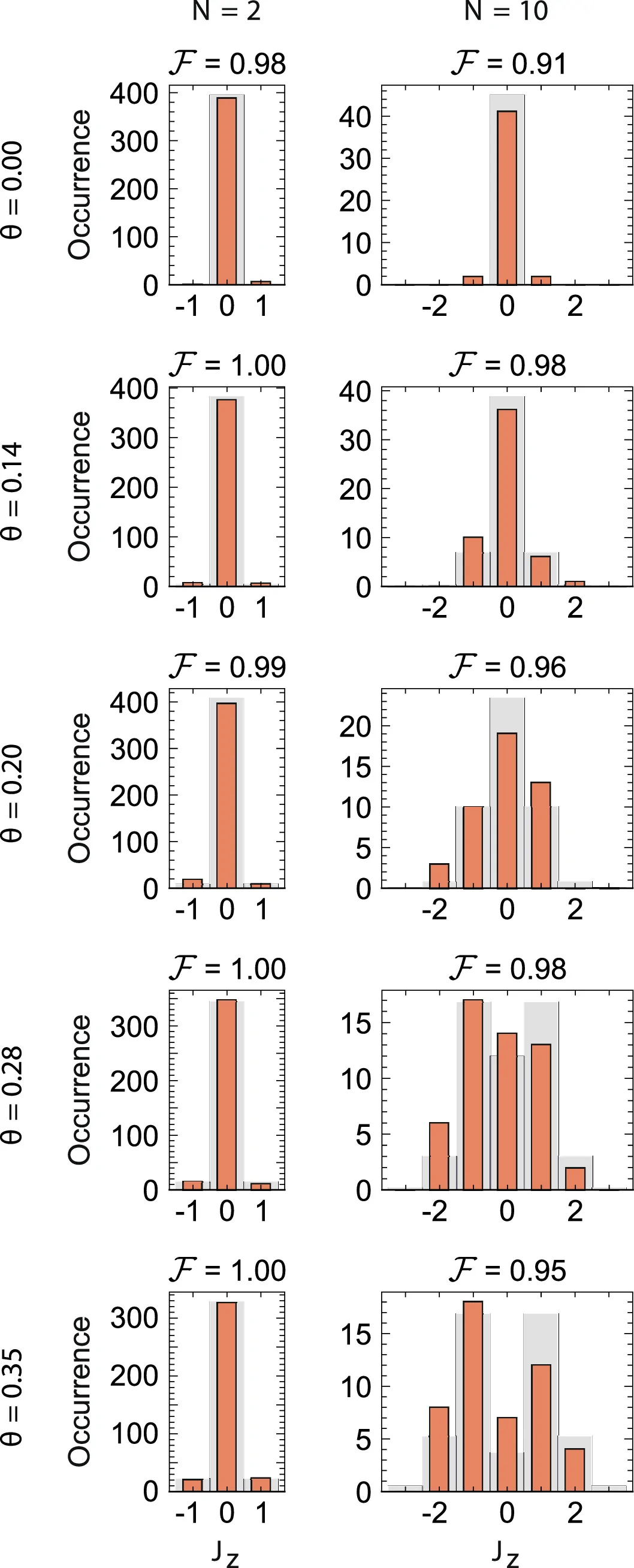
Increased rate of change due to larger Fisher information. The probabilities of different *J*_*z*_ values change with the rotation angle *θ* (different rows). The measured occurrences (orange bars) very closely follow direct, model-free predictions (gray bars), indicated by fidelities ${\mathcal{F}}$ close to unity. The *J*_*z*_ distribution for the *N* = 10 state (right column) changes much faster than that of the *N* = 2 state (left column), an effect that is quantified by the state’s Fisher information *F*_*N*_. Source data

From the recorded occurrences for (*J*_*z*_; *N*) for the five small rotation angles *θ*, we estimated the Fisher information *F*_*N*_ when using the prepared *N*-atom twin-Fock states from the experimental probabilities ${p}_{\theta }^{\exp }({J}_{z};N)$, here normalized such that ${\sum }_{{J}_{z}}{p}_{\theta }^{\exp }({J}_{z};N)=1$. Measured occurrences for *N* = 2 and *N* = 10 are displayed in Extended Data Fig. 5. The data clearly show that the rate at which the measured experimental probabilities change with the rotation angle *θ* is much larger for the *N* = 10 atom state.

To estimate the uncertainties in the analysis, we performed a Monte Carlo resampling of the measurement occurrences, which we used to calculate the experimental probabilities ${p}_{{\theta }_{1,2}}^{\exp }({J}_{z};N)$. We assumed multinomial distributions with event probabilities given by ${p}_{{\theta }_{1,2}}^{\exp }({J}_{z};N)$ for the *N* + 1 possible outcomes of *J*_*z*_. Subsequently, we computed the Hellinger distance of the recomputed distributions according to equation (5) and obtained the final value ${d}_{{\rm{H}},\mathrm{fit}}^{2}({\theta }_{1},{\theta }_{2};N)$ as the mean and its uncertainty as the standard deviation.

For each possible choice of reference angle *θ*_1_, we fitted the obtained values using the quadratic function

30 $${d}_{{\rm{H}},\mathrm{fit}}^{\,2}({\theta }_{1},{\theta }_{2};N\,)=\frac{{F}_{N}({\theta }_{1})}{8}{({\theta }_{1}-{\theta }_{2})}^{2}+b,$$

where *F*_*N*_(*θ*_1_) and *b* are free fitting parameters.

Finally, we computed the weighted average of the Fisher information obtained for different *θ*_1_ as

31 $${\bar{F}}_{N}=\mathop{\sum }\limits_{{\theta }_{1}}\frac{{w}_{{\theta }_{1}}}{{\sum }_{\theta }{w}_{\theta }}{F}_{N}({\theta }_{1})\,{\rm{,\; with}}$$

32 $${w}_{{\theta }_{1}}={\left(\frac{1}{\Delta {F}_{N}({\theta }_{1})/{F}_{N}({\theta }_{1})}\right)}^{2}.$$

Here the weights ${w}_{{\theta }_{1}}$ were computed from the relative uncertainty of *F*_*N*_(*θ*_1_). For the uncertainty of ${\bar{F}}_{N}$, we used the averaged uncertainties of the fitting parameters *F*_*N*_(*θ*_1_) rather than the standard error of the mean, as the five values *F*_*N*_(*θ*_1_) are not statistically independent.

We note that the resampling method introduced a statistical bias. As ${d}_{{\rm{H}},\mathrm{fit}}^{2}({\theta }_{1},{\theta }_{2};N)$ is a convex function, this bias was positive. Comparing the obtained mean values with those directly calculated from the measured experimental probabilities, we see that the bias depends only on the available sample sizes and not directly on *θ*. As these sample sizes were almost independent of *θ*, the free fitting parameter *b* can account for the introduced bias.

We further note that the Hellinger method itself is also affected by a statistical bias^53^, especially when the sample sizes are small. To quantitatively determine the effect, we employed the probabilistic noise estimation model to predict probability distributions for all rotation angles *θ*. From these probabilities, we calculated the Hellinger distance directly, without any form of random sampling or rounding to integer occupation numbers. Thus, no statistical bias was expected. Employing the same fitting techniques as for the measured data, the model gives a scaling $\frac{{N}^{1.81}}{2}+N$. Adding the fourth-order Taylor expansion term $-(\frac{1}{256}{F}_{N}^{2}-\frac{1}{192}{F}_{N}){\theta }^{4}$ to the fitting function ${d}_{{\rm{H}},\mathrm{fit}}^{2}$, as expected for twin-Fock states, resulted in $\frac{{N}^{1.87}}{2}+N$. We further note that the measurement point for *θ* = 0.35 rad and *N* = 14 atoms lies outside the range in which ${d}_{{\rm{H}}}^{2}({\theta }_{1},{\theta }_{2};N)$ can be approximated by a Taylor expansion, as there is a non-differentiable point at *θ* = 0.321 rad. Neglecting this data point in the fit of *F*_*N*_ yielded a scaling of $\frac{{N}^{2.01}}{2}+N$. Thus, our measurements are compatible with the *N*^2^ scaling of the ideal twin-Fock state.

### Calculating uncertainties

Unless stated otherwise, the error bars throughout this article represent the standard errors. For further details of our calculations of uncertainties, see ref. ^50^.

For Fig. 4 and Extended Data Fig. 4, the uncertainties were calculated using a Monte Carlo resampling approach. This method is analogous to that used in our Fisher information analysis. For each number of atoms *N*, we resampled the occurrences of the *J*_*z*_ values (without HOM coupling) and *J*_*x*_ values (after HOM coupling). This resampling used multinomial distributions, where the sample sizes and probabilities were derived from the measured data, specifically ${p}_{\theta }^{\exp }({J}_{z};N)$, which was normalized such that ${\sum }_{{J}_{z}=-N/2}^{N/2}{p}_{\theta }^{\exp }({J}_{z};N)=1$. From each obtained sample (indexed by *i* = 0, 1, …, 10,000), we calculated value pairs ${\{\langle {J}_{x}^{2}+{J}_{y}^{2}\rangle ,\langle {\varPi }_{z}\rangle \}}_{i}$ for equation (4) and ${\{\langle {J}_{x}^{2}+{J}_{y}^{2}\rangle ,{(\Delta {J}_{z})}^{2}\}}_{i}$ for equations (22) and (23). We then computed two samples {*k*_*i*_} of entanglement-depth values: one based on parity (for Fig. 4) and one based on the variance of *J*_*z*_ (for Extended Data Fig. 4). The displayed *k* values are the average of the {*k*_*i*_}, and their uncertainties are displayed using upper and lower standard deviations, as described below in equation (33). Extended Data Table 2 shows the minimally verified entanglement depths for confidence regions of 68% and 95%, that is the largest possible integer *k*_min_ such that ${k}_{i}\ge {k}_{\min }$ still holds for at least 68% or 95% of the samples.

The new entanglement-depth criterion, equation (4), is applicable only for *k* ≥ *N*/2. For 1.31% (*N* = 10) and 6.81% (*N* = 12) of the resampled value pairs, the criterion could not detect entanglement. In these cases, we used the criterion given by equations (22) and (23) to detect entanglement.

For the asymmetric error bars in Fig. 4 and Extended Data Fig. 4, we used the definitions for the upper and lower variances:

33 $$\begin{array}{rcl}{({\Delta }_{+}x)}^{2} & = & \frac{2}{M}\mathop{\sum }\limits_{n:{x}_{n}\ge \langle x\rangle }{({x}_{n}-\langle x\rangle )}^{2},\\ {({\Delta }_{-}x)}^{2} & = & \frac{2}{M}\mathop{\sum }\limits_{n:{x}_{n} < \langle x\rangle }{({x}_{n}-\langle x\rangle )}^{2},\end{array}$$

where *x*_*n*_ for *n* = 1, 2, …, *M* are a set of values and 〈*x*〉 is the average. With these definitions, for the variance

34 $${(\Delta x)}^{2}=\frac{{({\Delta }_{+}x)}^{2}+{({\Delta }_{-}x)}^{2}}{2}$$

holds.

## Online content

Any methods, additional references, Nature Portfolio reporting summaries, source data, extended data, supplementary information, acknowledgements, peer review information; details of author contributions and competing interests; and statements of data and code availability are available at https://doi.org/10.1038/s41567-026-03302-7.

## Supplementary information

Source Data Fig. 2–5 and Source Data Extended Data Fig. 2–5 Camera image evaluation results for the 7 × 3,816 repetitions of the seven measurement scenarios presented in the paper (see Extended Data Fig. 1).

## Figures and Tables

**Extended Data Table 1:**
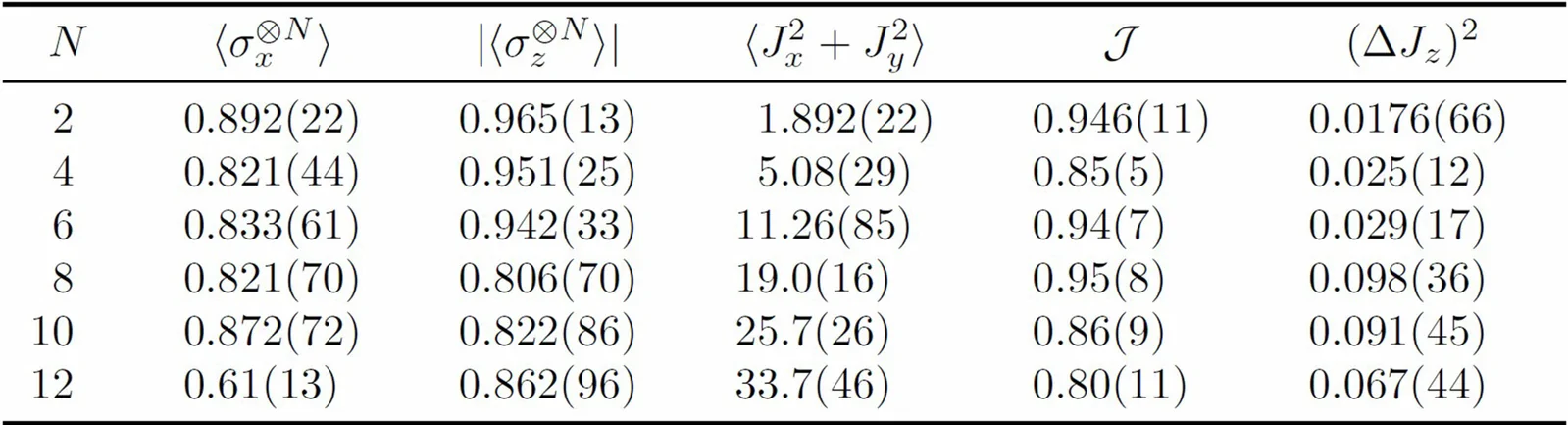
Measurement results for various particle numbers

The uncertainties denote one standard deviation.

**Extended Data Table 2:**
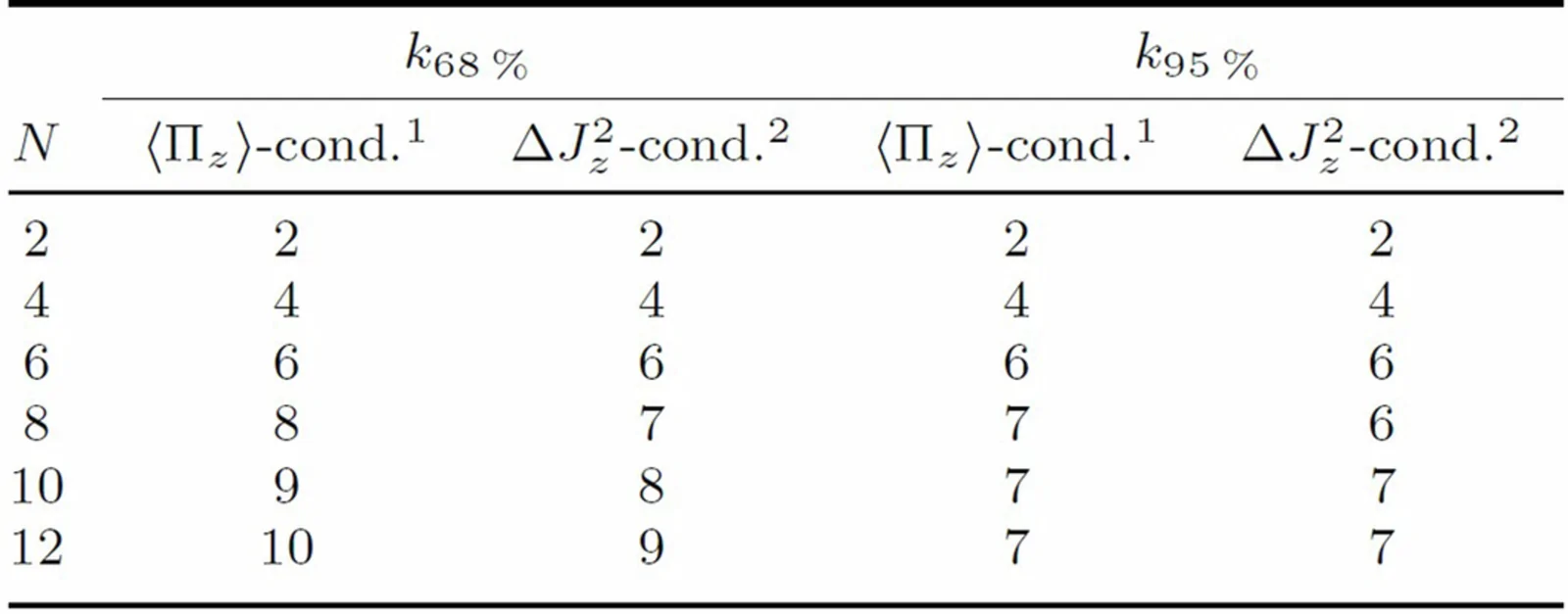
Minimally verified entanglement depth for confidence regions of 68% and 95%

^1^ According to equation (4), with equation (22) and equation (23) only as fall-back options (Methods, section *Calculation of uncertainties*).

^2^ According to equation (22) and equation (23).

## Data Availability

The raw fluorescence signal data, along with all other datasets used to generate the plots in this paper, are available via the LUIS data repository at https://doi.org/10.25835/b77m3ufp (ref. ^74^).
